# *speG* Is Required for Intracellular Replication of *Salmonella* in Various Human Cells and Affects Its Polyamine Metabolism and Global Transcriptomes

**DOI:** 10.3389/fmicb.2017.02245

**Published:** 2017-11-15

**Authors:** Shiuh-Bin Fang, Ching-Jou Huang, Chih-Hung Huang, Ke-Chuan Wang, Nai-Wen Chang, Hung-Yin Pan, Hsu-Wei Fang, Ming-Te Huang, Ching-Kuo Chen

**Affiliations:** ^1^Division of Pediatric Gastroenterology and Hepatology, Department of Pediatrics, Shuang Ho Hospital, Taipei Medical University, Taipei, Taiwan; ^2^Department of Pediatrics, School of Medicine, College of Medicine, Taipei Medical University, Taipei, Taiwan; ^3^Master Program for Clinical Pharmacogenomics and Pharmacoproteomics, College of Pharmacy, Taipei Medical University, Taipei, Taiwan; ^4^Graduate Institute of Biochemical and Biomedical Engineering, National Taipei University of Technology, Taipei, Taiwan; ^5^Graduate Institution of Engineering Technology-Doctoral, National Taipei University of Technology, Taipei, Taiwan; ^6^Institute of Biomedical Engineering and Nanomedicine, National Health Research Institutes, Zhunan, Taiwan; ^7^Department of Surgery, Shuang Ho Hospital, Taipei Medical University, Taipei, Taiwan; ^8^Department of Surgery, School of Medicine, College of Medicine, Taipei Medical University, Taipei, Taiwan

**Keywords:** *speG*, polyamine, transcriptome, *Salmonella* Typhimurium, RNA microarray, flagella, motility, intracellular replication

## Abstract

The *speG* gene has been reported to regulate polyamine metabolism in *Escherichia coli* and *Shigella*, but its role in *Salmonella* remains unknown. Our preliminary studies have revealed that *speG* widely affects the transcriptomes of infected *in vitro* M and Caco-2 cells and that it is required for the intracellular replication of *Salmonella enterica* serovar Typhimurium (*S*. Typhimurium) in HeLa cells. In this study, we demonstrated that *speG* plays a time-dependent and cell type-independent role in the intracellular replication of *S*. Typhimurium. Moreover, high-performance liquid chromatography (HPLC) of four major polyamines demonstrated putrescine, spermine, and cadaverine as the leading polyamines in *S*. Typhimurium. The deletion of *speG* significantly increased the levels of the three polyamines in intracellular *S*. Typhimurium, suggesting the inhibitory effect of *speG* on the biosynthesis of these polyamines. The deletion of *speG* was associated with elevated levels of these polyamines in the attenuated intracellular replication of *S*. Typhimurium in host cells. This result was subsequently validated by the dose-dependent suppression of intracellular proliferation after the addition of the polyamines. Furthermore, our RNA transcriptome analysis of *S*. Typhimurium SL1344 and its *speG* mutant outside and inside Caco-2 cells revealed that *speG* regulates the genes associated with flagellar biosynthesis, fimbrial expression, and functions of types III and I secretion systems. *speG* also affects the expression of genes that have been rarely reported to correlate with polyamine metabolism *in Salmonella*, including those associated with the periplasmic nitrate reductase system, glucarate metabolism, the phosphotransferase system, cytochromes, and the succinate reductase complex in *S*. Typhimurium in the mid-log growth phase, as well as those in the *ilv*–*leu* and histidine biosynthesis operons of intracellular *S*. Typhimurium after invasion in Caco-2 cells. In the present study, we characterized the phenotypes and transcriptome effects of *speG* in *S*. Typhimurium and reviewed the relevant literature to facilitate a more comprehensive understanding of the potential role of *speG* in the polyamine metabolism and virulence regulation of *Salmonella*.

## Introduction

Non-typhoidal *Salmonella* are important pathogens that cause a wide spectrum of diseases and considerable morbidity and mortality in humans and animals worldwide (Hohmann, 2001). *Salmonella* are invading and intracellularly replicating bacteria (Dougan et al., 2011). Host cell invasion (Pace et al., 1993) and intracellular replication (Leung and Finlay, 1991) are essential for the pathogenesis of *Salmonella enterica* serovar Typhimurium (*S*. Typhimurium). More than 100 virulence-associated genes have been discovered among the approximately 4,500 genes present in the genome of *S*. Typhimurium (McClelland et al., 2001). Most virulence genes are clustered in at least 23 *Salmonella* pathogenicity islands (SPIs) distributed on the *Salmonella* chromosome (Espinoza et al., 2017), including 11 common SPIs in *S*. Typhimurium and *S*. Typhi (Sabbagh et al., 2010). The most commonly studied SPIs, SPI-1 and SPI-2, encode type III secretion systems (T3SSs), which can translocate effector proteins into host cells or secrete them into the extracellular environment to manipulate host cell physiology and biochemistry (Coburn et al., 2007). SPI-1 genes facilitate bacterial invasion in non-phagocytic cells and uptake into phagocytic cells in the early phase of infection (Bueno et al., 2010). By contrast, SPI-2 genes account for intracellular survival and the evasion of the oxidase defense system of host cells, particularly in the systemic phase of salmonellosis (Coburn et al., 2007). The SPI-2 T3SS is essential for bacterial intracellular replication in *Salmonella*-containing vacuoles in host cells through the translocation of approximately 30 SPI-2 T3SS effector proteins into the host endomembrane system and cytosol (Figueira and Holden, 2012). However, the physiological relevance of SPI-2 T3SS effectors and the effects of their coordination on SPI-2 T3SS-mediated intracellular replication remain unclear (Helaine et al., 2010). Until now, SPI-2 T3SS genes associated with the intracellular replication of *Salmonella* have been mostly reported in phagocytic cells (Helaine et al., 2010; Figueira and Holden, 2012; Figueira et al., 2013). A few studies have demonstrated that SPI-1 T3SS genes are required for intracellular replication in human cervical epithelial cells (Steele-Mortimer et al., 2002) and 3-dimensional colonic epithelial cells (Radtke et al., 2010). Furthermore, additional studies have reported that auxotrophic mutations in aromatic amino acid metabolism and purine biosynthesis attenuated the intracellular replication of *S*. Typhimurium in various cell lines, including Madin–Darby canine kidney epithelial cells, human cervical HeLa cells, and intestinal epithelial Caco-2 and T-84 cells (Leung and Finlay, 1991; Holzer and Hensel, 2012). However, virulence genes involved in the intracellular replication of *Salmonella* in human non-phagocytic epithelial cells have not been thoroughly investigated.

Following the isolation of 10 auxotrophic replication-defective mutants from the 45,000 transposon mutants of *S*. Typhimurium in 1991 (Leung and Finlay, 1991), large-scale screening studies using high-throughput technologies, including libraries of transposon mutants and transcriptomic analysis, have identified previously unreported genes that are required for intracellular replication in non-phagocytic cells. The intracellular proliferation of *S*. Typhimurium occurs in cultured epithelial and macrophage cells but not in normal fibroblast cells (Martinez-Moya et al., 1998; Cano et al., 2001; Nunez-Hernandez et al., 2013). Thus, 50,000 independent transposon Mu*d*J-generated mutants derived from wild-type *S*. Typhimurium were selected in rat kidney fibroblasts after 72-h intracellular incubation. Genome analysis of the non-proliferating intracellular mutants revealed that a novel gene, *igaA*, suppresses their growth within fibroblasts (Cano et al., 2001). Meanwhile, mutations in *phoQ, rpoS, slyA*, and *spvR*, which have been demonstrated to be essential for the *in vivo* intracellular proliferation of *Salmonella*, resulted in the attenuation of intracellular bacterial growth in fibroblasts. This suggested that the PhoP–PhoQ two-component system is a negative regulator of bacterial growth in fibroblasts and so are the different phenotypes of these genes in diverse cell types (Cano et al., 2001). A recent genome-wide study conducted using the expression profiling of non-growing wild-type *S*. Typhimurium collected at 24 h postinfection in the same rat fibroblasts revealed that approximately 2% of the *S*. Typhimurium genome was differentially expressed in non-proliferating intracellular bacteria. This included the 98 genes involved in metabolic reprogramming for microaerophilic conditions, the induction of virulence plasmid genes, the upregulation of SPI-1 and SPI-2, and the shutdown of chemotaxis and flagellation (Nunez-Hernandez et al., 2013). Similarly, the transcriptome showed activated functions of PhoP–PhoQ-regulating PagN, PagP, and VirK in dormant intracellular bacteria after sensing vacuolar acidic pH for preventing intracellular overgrowth (Nunez-Hernandez et al., 2013). Another non-phagocytic cell line, HeLa cell line, has been extensively used to study intracellular replication of not only *Salmonella* spp. but also of enteroinvasive *Escherichia coli* (*E. coli*) and *Yersinia* spp. (Small et al., 1987; Leung and Finlay, 1991; Hautefort et al., 2008). The microarray analysis of time-dependent changes in *Salmonella* gene expression in HeLa cells and J774A.1 murine macrophages demonstrated the upregulation of *iro, mgtBC*, and *pstACS*; genes for iron, magnesium, and phosphate uptake; and SPI-2 (Hautefort et al., 2008). The invasion-associated SPI-1 and flagellar genes are upregulated in epithelial cells at 6 h postinfection when bacteria are intracellularly replicating but are constantly downregulated in J774A.1 murine macrophages (Hautefort et al., 2008). A recent study conducted using a mutational approach reported that the replication of *S*. Typhimurium in murine colonic epithelial cells requires glycosis and ubiquinone, but not an intact tricarboxylic acid cycle (TCA cycle), adenosine triphosphate (ATP) synthase, and fermentation (Garcia-Gutierrez et al., 2016). It remains unclear whether malate could be replenished by succinate or its precursors from non-phagocytic cells similar to phagocytes, although conversion from succinate to fumarate, from fumarate to malate, and from malate to both oxaloacetate and pyruvate in the TCA cycle are required for full virulence of *S*. Typhimurium in mice (Tchawa Yimga et al., 2006; Mercado-Lubo et al., 2008, 2009). So far, the virulence genes involved in the intracellular replication of *Salmonella* in human intestinal epithelial cells have not been thoroughly investigated.

Our preliminary study conducted using a library of 1,440 transposon mutants of *S*. Typhimurium to invade HeLa cell monolayers for 10 h and a high-throughput genome-wide analysis through transposon-directed insertion-site sequencing (Chaudhuri et al., 2013) identified *speG* as a gene essential for the intracellular replication of *Salmonella* in human epithelial cells (Fang, 2011). However, it remains unknown whether this result is applicable to other cells. The *speG* mutant of *S*. Typhimurium is a non-replicating strain in human cells and is thus a candidate vaccine vector for interacting with intestinal epithelial cells (Wang et al., 2016b). We used RNA microarrays to determine whether *S*. Typhimurium and *speG* affect the transcriptomes of two human intestinal epithelial cells and identified *speG*-regulated genes, including *KYL4, SCTR, IL6, TNF*, and *CELF4* in Caco-2 cells and *JUN, KLF6*, and *KCTD11* in *in vitro* M cells, which are specialized intestinal epithelial cells conferring host immunity (Wang et al., 2016b). However, it is unclear whether *speG* regulates the expression of other genes in *Salmonella* before and after bacterial invasion in human intestinal epithelium.

Until now, knowledge regarding *speG* has been obtained from studies mainly conducted in *E. coli* and *Shigella*, but rarely in *Salmonella*. *speG* is involved in polyamine metabolism and stress responses in bacterial pathogenesis. It encodes spermidine acetyltransferase (SAT), which catalyzes spermidine to acetylspermidine in *E. coli*. However, the *speG*-dependent acetylation of spermidine and the *speE*-dependent catabolization of cadaverine into aminopropyl cadaverine are not conserved in *Shigella* spp. (Barbagallo et al., 2011). The accumulation of spermidine is toxic for *E. coli* and reduces the viability of the *speG*-deficient mutant of *E. coli* at the late stationary growth phase. However, excessive spermidine can be inactivated by its *speG*-catalyzed acetylation to acetylspermidine, which is either stored or secreted by the cells (Fukuchi et al., 1995). The other spe genes, including *speB, speC, speE*, and *speF*, have been reported to contribute to the intracellular survival and replication of *S*. Typhimurium in human epithelial cells for 18 h (Jelsbak et al., 2012) and in macrophages for 21 h (Espinel et al., 2016). However, the role of *speG* in regulating polyamine metabolism and influencing the intracellular replication of *Salmonella* in human intestinal epithelial cells has been rarely investigated.

Polyamine composition and the predominant polyamine in *Salmonella* are unclear. Until now, our understanding of polyamines in bacteria has mainly been established through studies of *E*. *coli*. Putrescine, spermidine, spermine, and cadaverine are the major cellular polyamines essential for the normal cellular proliferation and growth of both prokaryotic and eukaryotic cells (Cohen, 1997; Shah and Swiatlo, 2008). The intracellular concentration of spermidine is much higher than that of putrescine in almost all bacteria, but 10 times lower than that of putrescine in *E. coli* (Cohen, 1997; Shah and Swiatlo, 2008). Spermine is only found in the presence of exogenous spermine in most bacteria, whereas cadaverine, typically absent in *E. coli*, is the least widespread of naturally occurring bacterial polyamines (Cohen, 1997). Putrescine constitutes the outer membrane of *S*. Typhimurium and *E. coli* (Koski and Vaara, 1991). However, whether putrescine is the predominant intracellular polyamine in *Salmonella*, similar to *E. coli*, requires further validation.

In this study, we examined whether the deletion of *speG* affects the intracellular proliferation of *S*. Typhimurium in various human cells. Subsequently, we studied the polyamine metabolism of *S*. Typhimurium and the effect of *speG* by quantifying the four major polyamines in extracellular and intracellular wild-type and *speG*-deleted strains. We verified whether the accumulation of polyamines suppresses the intracellular proliferation of *S*. Typhimurium. Moreover, we investigated how *speG* regulates the transcriptome of *S*. Typhimurium before and after invasion in human intestinal epithelium. Finally, we determined whether the deletion of *speG* affects the motility and flagellation of *S*. Typhimurium.

## Materials and methods

### Bacterial strains and culture conditions

The *S*. Typhimurium wild-type strain SL1344, its isogenic *speG*-deleted mutant Δ*speG*, the *speG*-complemented strain of Δ*speG* (Δ*speG*′), the *fliC*-deleted flagellin-deficient mutant Δ*fliC*, and *spaS*-deleted invasion-deficient SPI-1 mutant Δ*spaS* were used in this study. SL1344 (Mo et al., 2006) and Δ*spaS* (Buckley et al., 2010) were kindly provided by Prof. Duncan Maskell. The *S*. Typhimurium SL1344 genome has been completely sequenced, and its complete sequence and annotation are available in Genbank (accession numbers FQ312003 and HE654724-6). The mutants Δ*fliC* and Δ*spaS* were used as controls. Δ*speG* and Δ*fliC* were constructed using the lambda red recombinase-mediated integration of linear polymerase chain reaction (PCR) amplicons to replace the target gene with a kanamycin resistance gene cassette, as previously reported (Gust et al., 2003; Wang et al., 2016a,b). For generating the *speG*-complemented *S*. Typhimurium strain (Δ*speG*′), the *speG*-coding sequence was amplified using *speG*-specific primers (forward, 5′-ATCTTACTGCGCGGTGGGTT-3′, and reverse, 5′-GATGCAGGATAACTAAAAGGAAGTGTAAGGATACAGTATGA-3′) and cloned into the pBluescript II KS(−) vector in the EcoRV site. Subsequently, the cloned vector was digested by EcoRV and ligated with the apramycin resistance gene, *aac(3)IV*, which was then amplified using the *aac(3)IV* specific primers (forward, 5′-TACCACCGCTGGTAGCGGT-3′, and reverse, 5′-AAACCGGGCGCGGTGCGACTCTCCGTGACTACCGCGCCGCGACGCTGATCGTGCGGGAG-3′) in the same gene orientation. With 41 nucleotides at both ends homologous to the replacing kanamycin resistant gene, the fused *speG*-*aac(3)IV* DNA fragment was amplified using the primers (forward, 5′-GATGCAGGATAACTAAAAGGAAGTGTAAGGATACAGTATGA-3′, and reverse, 5′-AAACCGGGCGCGGTGCGACTCTCCGTGACTACCGCGCCGCGACGCTGATCGTGCGGGAG-3′), then transferred into the Δ*speG* mutant. The *speG* expression was restored following the same red recombination strategy as previously prescribed (Wang et al., 2016a). The Δ*speG*′ was maintained in LB broth supplemented with apramycin (50 μg/mL) at 37°C. This study was conducted in the Biosafety Level 2 Laboratory that had been approved by the Biosafety Committee of Taipei Medical University Shuang Ho Hospital (No. BSL-2-0001).

### *In vitro* cell cultures

Four human cell lines used in this study were purchased from Bioresource Collection and Research Center, Taiwan. HeLa cells (BCRC No. 60005, originally from ATCC CCL-2), which are an epithelial cell line of human cervical carcinoma, and LS174T cells (BCRC No. 60053, originally derived from ATCC CL-188), which are a human intestinal epithelial cell line of Caucasian Duke's type B colorectal adenocarcinoma, were cultured in 90% Dulbecco's modified Eagle medium (DMEM, 4,500 mg/L glucose; Gibco) complemented with 2 mM L-glutamine (Gibco) adjusted to contain 1.5 g/L sodium bicarbonate (Sigma), 0.1 mM non-essential amino acids (Gibco), 1 mM sodium pyruvate (Gibco), and 10% fetal bovine serum (FBS, Sigma). Furthermore, Caco-2 cells (BCRC No. 67001, originally from ATCC HTB-37), a human intestinal epithelial cell line of a Caucasian colon adenocarcinoma, were cultured in the same medium as that for HeLa cells, except for the substitution of 10% FBS with 20% FBS. THP-1 cells (BCRC No. 60430, originally from ATCC TIB-202), a cell line of human acute monocytic leukemia, were cultured in a suspension of 90% RPMI 1640 medium (Gibco) complemented with 2 mM L-glutamine (Gibco) adjusted to contain 1.5 g/L sodium bicarbonate, 2.5 g/L glucose, 10 mM 4-(2-hydroxyethyl)piperazine-1-ethanesulfonic acid (HEPES), 1 mM sodium pyruvate, 0.05 mM 2-mercaptoethanol (Gibco), and 10% FBS. For maintenance, these cells were grown in 75-cm^2^ flasks in humidified 5% CO_2_ at 37°C and were split in a 1:4 ratio of 0.25% trypsin–ethylenediaminetetra acetic acid (Gibco) before complete confluence.

For *in vitro* infection assays, these cells were seeded at a density of 5 × 10^5^ cells/well into 12-well plates and were maintained in humidified 5% CO_2_ at 37°C. The cell culture medium was replaced every other day when the cells were incubated for 3, 4, and 5 days until a complete confluence of the cell monolayers was achieved at a density of approximately 1 × 10^6^ cells/well for HeLa cells, 8 × 10^5^ cells/well for Caco-2 cells, and 2 × 10^6^ cells/well for LS 174T cells, respectively. Meanwhile, THP-1 cells in suspension were seeded at a density of 2 × 10^6^ cells/well into 12-well plates and were incubated with 10 ng/well phorbol myristate acetate (Sigma) for 24 h to induce differentiation into adherent macrophages for further assays. The medium in each well of the cell monolayer was replaced with their corresponding complete medium without FBS 1 h before assays.

### Bacterial intracellular replication assay

HeLa cell monolayers were infected with overnight cultures of *S*. Typhimurium SL1344, Δ*speG*, and Δ*speG*′ [multiplicity of infection (MOI) = 5] in duplicate wells for each bacterial strain and were incubated in 5% CO_2_ at 37°C for 2 h. After washing three times with phosphate-buffered saline (PBS), the infected cells were incubated in FBS-free DMEM supplemented with gentamicin (100 μg/mL) for 1 h; thereafter, the cells were washed with PBS three times to kill extracellular bacteria. At this point, one set of the cells infected with the three *S*. Typhimurium strains was lysed with 1% Triton X-100 to generate output pool A, which represents invading bacteria. The other two sets of the cells infected with the *S*. Typhimurium strains were incubated for an additional 7 and 10 h in FBS-free DMEM supplemented with low-dose gentamicin (10 μg/mL) to allow intracellular infections to continue. The cells were subsequently washed with PBS three times and lysed with 1% Triton X-100 (Sigma) to generate output pools B1 and B2, respectively, which represent intracellularly replicating bacteria after incubation at different durations.

The prepared confluent HeLa, Caco-2, LS 174T, and THP-1 cells in 12-well plates were infected with overnight cultures of the three *S*. Typhimurium strains (MOI = 5) for 2 h and treated with gentamicin (100 μg/mL) for 1 h using the same protocol as that used for obtaining output pool A. After washing with PBS three times, the cells were incubated for an additional 15 h. Finally, the cells were lysed with 1% Triton X-100 to generate output pool B, which contained bacteria proliferated in the cells for a total of 18 h.

The infected cell monolayers were stained with trypan blue to confirm a viability of >95% in all the wells before cell lysis to obtain output pools B1, B2, and B. The intracellular bacterial counts respective to the initial inoculums in output pools A, B1, B2, and B were compared between the two recombinant strains and wild-type strain of *S*. Typhimurium by using the Student's *t*-test. The intracellular bacterial counts in all output pools are expressed as mean ± standard error colony-forming units (CFU) per inoculum of 10^7^ CFU. *p* < 0.05 was considered statistically significant.

### High-performance liquid chromatography quantification of four major polyamines in *S*. typhimurium SL1344 and Δ*speG* before and after invasion in Caco-2 cells

Before the experiment, overnight cultures of *S*. Typhimurium SL1344 and Δ*speG* were 1:100 diluted in Luria–Bertani (LB) broth and incubated with shaking at 225 rpm in 5% CO_2_ at 37°C for 3 h to generate mid-log cultures, which were considered as extracellular bacteria. The prepared confluent Caco-2 cells in the 75-cm^2^ flasks were infected with mid-logarithmic cultures of *S*. Typhimurium SL1344 and Δ*speG* (MOI = 5) for a total of 18 h by using the same protocol as that for obtaining the output pool B in three independent experiments. The Caco-2 cells infected by these two strains of *S*. Typhimurium were lysed with 1% Triton X-100 to obtain the intracellular bacteria.

Next, polyamines in extracellular bacteria from the mid-log cultures of *S*. Typhimurium SL1344 and Δ*speG*, and the Caco-2 cell lysates containing intracellular bacteria of these two strains were extracted using trichloroacetic acid (TCA; Sigma). The mid-log cultures were centrifuged (4,000 × g) at 4°C for 10 min; the supernatants were removed, and the bacterial pellets were washed with PBS. The centrifugation and PBS washing protocols were performed twice, and the bacterial pellets were resuspended in lysis buffer [20 mM 3-(N-morpholino) propanesulfonic acid, pH 8.0, 10 mM NaCl, and 4 mM MgCl_2_]. The bacterial cells were lysed through ultrasonic vibration. Finally, 100 μL of 40% TCA was added to the bacterial lysates on ice for 5 min and centrifuged (13,000 × g) at 4°C for 3 min. The supernatants of both extracellular *S*. Typhimurium strains were decanted and stored at −20°C for high-performance liquid chromatography (HPLC) analysis.

The intracellular bacteria in the Caco-2 cell lysates were filtered using a 7-μm filter to remove the cell debris, and the pellets were resuspended in LB broth under shaking at 225 rpm in 5% CO_2_ at 37°C for 2 h to amplify the bacterial concentration of the two host cell-primed *S*. Typhimurium strains. The bacterial pellets were subsequently processed as TCA precipitation and polyamine extraction for the mid-log cultures of both *S*. Typhimurium strains. Finally, the supernatants of both intracellular strains were collected and stored at −20°C for HPLC.

The standard solutions of putrescine (purity: 99.9%; TCI), spermidine (purity: 99.7%; Fluka), spermine (purity: 99.8%; Fluka), and cadaverine (purity: 98.7%; 1,5-diaminopentane, Fluka), as well as the TCA-treated supernatants of extracellular and intracellular *S*. Typhimurium SL1344 and Δ*speG* were processed before HPLC analysis. Furthermore, 1 mL of 2 N NaOH (Sigma) and 10 μL of benzoyl chloride (Sigma) were added into each of the eight samples, vortexed for 30 s, and incubated with shaking at room temperature for 20 min to generate solutions of benzoyl-polyamines. Subsequently, 2 mL of saturated NaCl (Sigma) was added to the mixtures, which were then vortexed for 30 s. Finally, diethyl ether (Tedia) was added, and the mixtures were vigorously shaken. The solutions were stored at −20°C for 1 h. The diethyl ether phase of the solutions was then collected and incubated at 37°C, and the ether was removed through evaporation. Finally, individual samples were dissolved in 50% methanol (Echo Chemical) and stored at −20°C for 1 h.

**Figure 1:**
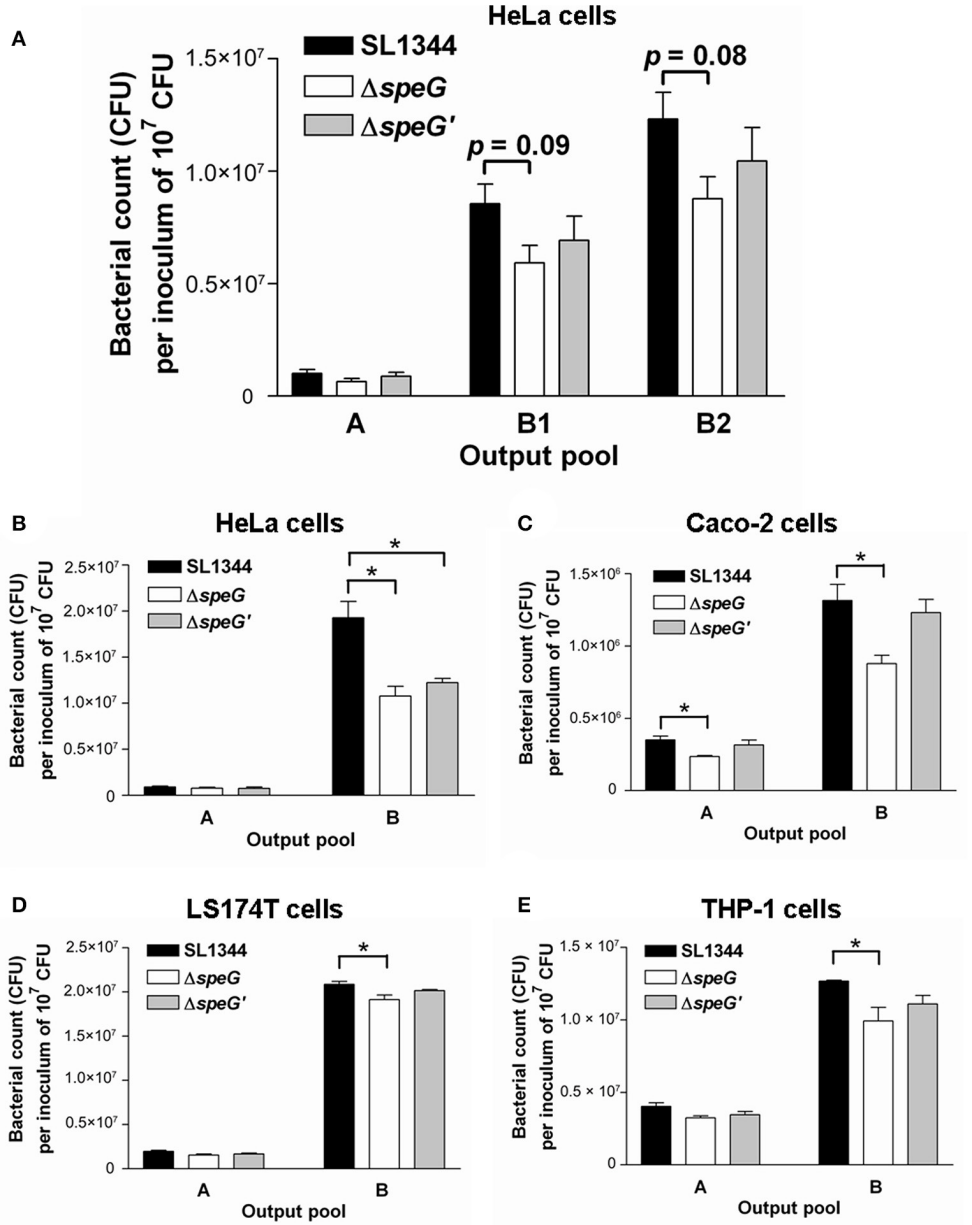
Intracellular bacterial replication assays yielded the intracellular bacterial concentrations of *S*. Typhimurium SL1344 and its Δ*speG* mutant at various time points after invasion in different human cell lines. Human cell lines were infected with *S*. Typhimurium wild-type SL1344, its Δ*speG* mutant, and the *speG*-complemented strain Δ*speG*′ (multiplicity of infection = 5) for 1 h. The extracellular bacteria were killed using gentamicin after another 2 h, and the intracellular bacteria in the infected cells were allowed to proliferate for additional 7 h (output pool B1), 10 h (output pool B2), or 15 h (output pool B). **(A)** *S*. Typhimurium Δ*speG* was non-significantly attenuated in output pools B1 and B2, in which intracellular bacteria in HeLa cells had been maintained for 10 and 13 h, respectively. **(B–E)** *S*. Typhimurium Δ*speG* was significantly attenuated in output pool B, in which intracellular bacteria had been maintained in HeLa, Caco-2, LS174T, and THP-1 cells for 18 h, as indicated by asterisks (^*^p < 0.05; *n* = 3).

The concentrations of TCA-treated extracellular and intracellular bacterial samples were determined through HPLC, as reported previously (Slocum et al., 1989; Lee et al., 2009). The published methods were modified (flow rate: 0.3 mL/min, absorbance detection: 254 nm, and temperature: 25°C) and applied during the mobile phase by using distilled water as buffer A and methanol as buffer B in the solvent gradient conditions as follows: initial, 50% B; 0–40 min, 50–70% B; 40–50 min, 70–50% B; and 50–70 min, 50% B. First, the standard solutions of 80 mM putrescine (purity 99.9%, TCI 110-60-1, 2 mM cadaverine (1,5-diaminopentane purity 98.7%, Fluka FL-33211), 8 mM spermidine (purity 99.7%, Fluka FL85561), and 40 mM spermine (purity 99.8%, Fluka FL85590) were 1:4 diluted and mixed for HPLC analysis to obtain the retention times of putrescine at 35 min, cadaverine at 40 min, spermidine at 54 min, and spermine at 65 min (Supplemetary Figure 1). Next, the five dilutions of the four polyamine standard solutions and the four TCA-treated bacterial samples were filtered using Hypersil ODS C18 Columns (Thermo Scientific) and injected into the HPLC apparatus (Waters 600 controller). Subsequently, the peak area values of the four polyamines in the bacterial samples and the serial dilutions of the standard solutions in the HPLC chromatogram were obtained and analyzed using the Autochro-3000 Chromatography Data System (Young Lin, Taiwan). Finally, the concentrations of the four polyamines in the TCA-treated bacterial samples were calculated by applying their peak area values to the regression equations derived from the five dilutions of the analyzed standard solutions (Supplementary Figure 2). These samples were bracketed with standards in five dilutions, including putrescine (1, 2, 5, 10, and 20 mM; peak areas between 40 and 1,200 mm^2^), cadaverine (0.1, 0.2, 0.5, 1, and 2 mM; peak areas between 100 and 3,500 mm^2^), spermidine (0.1, 0.2, 0.5, 1, and 2 mM; peak areas between 50 and 2,500 mm^2^), and spermine (1, 2, 5, 10, and 20 mM; peak areas between 40 and 1,500 mm^2^). The concentrations of the individual polyamines were compared between *S*. Typhimurium SL1344 and Δ*speG* before and after their invasion in Caco-2 cells by using the Student's *t*-test. Simiarly, the polyamine concentrations of extracellular and intracellular bacteria were also compared in *S*. Typhimurium SL1344 and Δ*speG*, respectively. The polyamine concentrations are expressed as mean ± standard error (mM per 10^9^ bacteria). *p* < 0.05 was considered statistically significant.

### Polyamine suppression assay

By using the same protocol as that used for obtaining output pool B, confluent Caco-2 cells in 12-well plates were infected with overnight cultures of *S*. Typhimurium SL1344, and the infected cells were treated with putrescine (625 and 312.5 μM), spermine (375 and 187.5 μM), and cadaverine (125 and 62.5 μM) or left untreated for 15 h. After 18-h incubation, the intracellular bacterial numbers from the treated Caco-2 cells were calculated as prescribed in the bacterial intracellular replication assays and compared between the treated and untreated groups by using the Student's *t*-test. The data are expressed as mean ± standard error CFU per inoculum of 10^7^ CFU. *p* < 0.05 was considered statistically significant.

### RNA microarrays of *S*. typhimurium SL1344 and Δ*speG* before and after invasion in Caco-2 cells

Confluent Caco-2 cells in the 75-cm^2^ flasks were infected with overnight cultures of *S*. Typhimurium SL1344 or Δ*speG* (MOI = 5) in two independent experiments by using the same protocol as that used for obtaining the output pool B. After 18-h incubation, the infected cells were lysed with 1% Triton X-100, and the cell lysates were passed through a 3-μm filter to remove the cell debris. After centrifugation at 800 × g for 10 min and the removal of supernatants, the bacterial pellets were washed with PBS to obtain the intracellular bacteria. The extracellular bacteria from the overnight cultures and the intracellular bacteria from the aforementioned processing of *S*. Typhimurium SL1344 and Δ*speG* were dissolved in TRIzol (Gibco) for isolating the total RNA according to the manufacturer's instruction. The purity of the RNA samples was validated using the ratio of absorbance at 260 and 280 nm, as well as the RNA integrity number determined using Bioanalyzer 2100 (Agilent Technology) with an RNA 6000 Nano LabChip kit (Agilent).

*In vitro* transcription was performed as previously described (Lee et al., 2009). Briefly, the total RNA samples were reverse transcribed to cDNAs and subsequent cRNAs, which were amplified and labeled with Cy3 (CyDye, Agilent). The Cy3-labeled cRNAs were subsequently fragmented to an average size of 50–100 nucleotides, pooled, and hybridized to Agilent Technologies custom *Salmonella* GE 8 × 15K microarray that had been tiled with 4,631 gene probes of *S*. Typhimurium SL1344. After washing the array chips and drying them through nitrogen gun blowing, the microarrays were scanned with an Agilent microarray scanner at 535 nm for Cy3-CTP. The scanned images were quantified and analyzed using Feature Extraction 10.5.1.1 software (Agilent). The background values were corrected using the spatial detrend surface value and were normalized by quantile. Finally, the gene expression in each array group was analyzed using the DAVID database (https://david.ncifcrf.gov/). A heap map with genes in each group that showed more than two-fold upregulation or downregulation was constructed based on their normalized values by using GeneSpring multiomic analysis software (Agilent). The microarray data has been deposited in GEO (http://www.ncbi.nlm.nih.gov/geo/) and is accessible via the GEO Accession Number GSE102885.

The transcriptomes derived from *S*. Typhimurium SL1344 and Δ*speG* were compared before and after their invasion to Caco-2 cells by using the Student's *t*-test. The data were expressed as mean log2 fold change relative to *S*. Typhimurium SL1344. *p* < 0.05, with a fold change of >1 log2 or <−1 log2 was considered statistically significant.

### Quantitative real-time polymerase chain reaction for confirmation of RNA analysis

Pairs of oligonucleotide primers specific to the selected genes identified from the microarrays and the housekeeping gene 16s (Table 1) were designed using Primer3 and BLAST (http://www.ncbi.nlm.nih.gov/tools/primer-blast/). The total RNAs of extracellular and intracellular *S*. Typhimurium SL1344 and Δ*speG* were isolated from their mid-log cultures and lysates of the infected Caco-2 cells by using the Total RNA Miniprep Purification Kit (Genemark, Taichung, Taiwan) according to the manufacturer's instruction. The total RNA samples were purified, and the residual DNA was eliminated using RNase-free DNase I (NEB, Beverly, MA, USA). Next, 0.1 μg of RNA was reverse transcribed to cDNA by using the Transcriptor High Fidelity cDNA Synthesis Kit (Roche Applied Science, Mannheim, Germany), according to the manufacturer's instruction. By using the Bio-Rad C100 Real-Time PCR System, quantitative real-time PCR (qRT-PCR) was performed in triplicate in a reaction volume of 25-μL solution containing 0.2 μM of primer pairs, 12.5 μL of iQ SyBr green supermix (BioRad), 9.5 μL of distilled H_2_O, and 1 μL of cDNA. The reaction solutions were heated at 95°C for 3 min and amplified for 40 cycles of 95°C for 15 s, 50°C for 30 s, and 72°C for 30 s. The mRNA transcription levels were determined using the ^ΔΔ^Ct method, as previously described (Wang et al., 2016b), and the expression of 16s ribosomal RNA was considered for normalization. The mRNA expression of the selected genes in extracellular and intracellular *S*. Typhimurium Δ*speG* mutant was compared with that of the same genes in the corresponding extracellular and intracellular *S*. Typhimurium SL1344 by using the Student's *t*-test. The data were expressed as mean ± standard error log2 fold change relative to *S*. Typhimurium SL1344. *p* < 0.05 was considered statistically significant.

**Table 1:** Primers used for qRT-PCR.

**Primer name (F: forward, R: reverse)**	**Sequence (5′ → 3′)**	**Product size (base pairs)**	**Description**
*flhA*-F	TGATGGTGCTGCCGCTACCT	124	Flagellar gene
*flhA*-R	GGAAACGCGGCAAAATCCA	124	Flagellar gene
*flhB*-F	GTTCGGCGGCGAGTCGTTAG	175	Flagellar gene
*flhB*-R	AAGCGCCACCAGCACCACG	175	Flagellar gene
*fliP*-F	TTCTCTGGCGGGTCTGTGGC	149	Flagellar gene
*fliP*-R	TCGCCGGCAGAAACGTCAGC	149	Flagellar gene
*fliQ*-F	TGATGATGGGCACCGAGGC	183	Flagellar gene
*fliQ*-R	ACGGGCCGGCAACGATAATT	183	Flagellar gene
*flgI*-F	ACCAGACGACCCAGACGCC	160	Flagellar gene
*flgI*-R	ATCGTTTGTCCCTGTCGCGC	160	Flagellar gene
*flgH*-F	AGGATGCGCCTGGATACCCG	183	Flagellar gene
*flgH*-R	ACAATCGTGAGCGTATCGCCG	183	Flagellar gene
*fimC*-F	TCGGCGGCGAAGCAGACTT	183	Type-1 fimbrial gene
*fimC*-R	CGGTTGCCCGGCGTAGAT	183	Type-1 fimbrial gene
*fimD*-F	ACCCTGCGTTTTTGTCGGCG	186	Type-1 fimbrial gene
*fimD*-R	GCATCAGGCCGCCGGATTTA	186	Type-1 fimbrial gene
*fimI*-F	TGCGCAGCCGGTAATGGTG	149	Type-1 fimbrial gene
*fimI*-R	CGAAGCTGCCAGGACCGGT	149	Type-1 fimbrial gene
*fimW*-F	TCTGGCGAATCAATGGCAGC	149	Type-1 fimbrial gene
*fimW*-R	TCGCCCGTAGCTGATGTTGG	149	Type-1 fimbrial gene
*ilvC*-F	AAATCGGCGAGCAGGAGTA	243	*ilv*–*leu* operon
*ilvC*-R	CGCAAGCGTAAGAGAACAG	243	*ilv*–*leu* operon
*leuD*-F	GCTTCGCCGACATCTTCTA	159	*ilv*–*leu* operon
*leuD*-R	TATCGCCTGCTTTCACCAC	159	*ilv*–*leu* operon
*hisG*-F	GTCTGGTAATGGATGGCGT	237	Histidine operon
*hisG*-R	GGTAGCGTTTGAGGAGGTG	237	Histidine operon
SL1344_2430-F	CGTCTGCGCATTAAGTTTC	153	Putative cobalamin adenosyltransferase
SL1344_2430-R	ATCGGGCTTTTTCACCACC	153	Putative cobalamin adenosyltransferase
*smvA*-F	TCTCCGCGTCTATCCGTCT	221	Methyl viologen resistance protein
*smvA*-R	GCTGAACCACATCCCTACC	221	Methyl viologen resistance protein
*pyrE*-F	AACCGCAAAGAGGCAAAAG	163	Orotate phosphoribosyltransferase
*pyrE*-R	TCAGTACGCCAGCCAGAGT	163	Orotate phosphoribosyltransferase
*speG*-F	GTAAATCCGTAATCCATCGC	248	Spermidine N1-acetyltransferase
*speG*-R	CGTTACTGGTTTGAAGAGCC	248	Spermidine N1-acetyltransferase
16S-F	TCGTCCAATGACCGGCAAGC	190	Housekeeping gene 16S
16S-R	TTCCTGCTGGCGCTGGAACT	190	Housekeeping gene 16S

### Bacterial motility assays

Semisolid LB agar plates containing 0.3% agar were used for performing the bacterial motility assay. Ten microliters of overnight cultures of *S*. Typhimurium SL1344, Δ*speG*, Δ*speG*′, Δ*spaS*, and Δ*fliC* were inoculated on the centers of the semisolid LB agar plates, followed by incubation at 37°C for 6 h. In this assay, the flagellated non-invasive SPI-1 mutant *S*. Typhimurium Δ*spaS* was considered the positive control, whereas the flagella-deficient mutants *S*. Typhimurium Δ*fliC* was used as the negative control. Furthermore, 10 μL of overnight cultures of *S*. Typhimurium SL1344 were inoculated on the centers of the semisolid LB agar plates supplemented with putrescine (625 and 312.5 μM), spermine (375 and 187.5 μM), cadaverine (125 and 62.5 μM), spermidine (6 and 3 μM), or left without any polyamine, and all the plates were incubated at 37°C for 6 h. The intensity of bacterial motility was determined by the diameters of bacterial growth zones. All the above assays were performed in three independent experiments.

### Transmission electron microscopy

*S*. Typhimurium SL1344 and Δ*speG* were visualized through transmission electron microscopy after negative staining. The overnight bacterial cultures were centrifuged at 5,000 rpm for 20 min, washed with distilled water twice, and fixed with 4% paraformaldehyde for 10 min. The bacteria on the grids were washed with distilled water twice, negatively stained with 2% uranyl acetate for 30 s, and subsequently rinsed with distilled water thrice. Finally, the samples were observed under a transmission electron microscope (FEI Tecnai G2 F20 S-Twin FEG).

## Results

### *speG* affects the intracellular replication of *S*. typhimurium in human cell lines

To confirm whether *speG* affects the intracellular proliferation of *S*. Typhimurium in infected human epithelial cells with time, HeLa cells were infected with *S*. Typhimurium SL1344 and Δ*speG* for 10, 13, and 18 h after killing extracellular bacteria during the third hour postinfection by performing gentamicin protection assays. Although *S*. Typhimurium Δ*speG* showed non-significant attenuation in bacterial proliferation in HeLa cells after 10 and 13 h postinfection (Figure 1A), intracellular *S*. Typhimurium Δ*speG* was significantly attenuated in output pool B in HeLa cells at 18 h postinfection, but not in output pool A at 3 h postinfection (1.1 × 10^7^ vs. 1.9 × 10^7^ CFU/well, *p* < 0.05; Figure 1B). Moreover, to investigate whether such a phenomenon existed in different cell types, the same *in vitro* intracellular replication assays were performed in Caco-2, LS174T, and THP-1 cells. We observed that the intracellular concentrations of *S*. Typhimurium Δ*speG* significantly decreased in LS174T cells (1.9 × 10^7^ vs. 2.1 × 10^7^ CFU/well, *p* < 0.05; Figure 1D) and THP-1 cells (1.0 × 10^7^ vs. 1.3 × 10^7^ CFU/well, *p* < 0.05; Figure 1E) compared with those of *S*. Typhimurium SL1344. The results revealed that *S*. Typhimurium Sl1344 and Δ*speG* invaded HeLa, LS174T, and THP-1 cells in similar concentrations, but the deletion of *speG* significantly attenuated the intracellular concentration of *S*. Typhimurium in these three cell lines after 18 h. Intracellular *S*. Typhimurium Δ*speG* significantly attenuated in both output pools A and B in Caco-2 cells (3.5 × 10^5^ vs. 2.4 × 10^5^ CFU/well and 1.3 × 10^6^ vs. 8.8 × 10^6^ CFU/well, respectively, *p* < 0.05; Figure 1C), with significantly higher attenuation of *S*. Typhimurium Δ*speG* than of *S*. Typhimurium SL1344 in output pool B than in output pool A (9.6 × 10^5^ vs. 6.4 × 10^5^ CFU/well, *p* < 0.05; Figure 1C). This suggested that the knockout of *speG* decelerated the intracellular amplification of *S*. Typhimurium in Caco-2 cells.

### Putrescine, spermine, and cadaverine are the major polyamines in *S*. typhimurium, regardless of *speG* and internalization in Caco-2 cells

**Figure 2:**
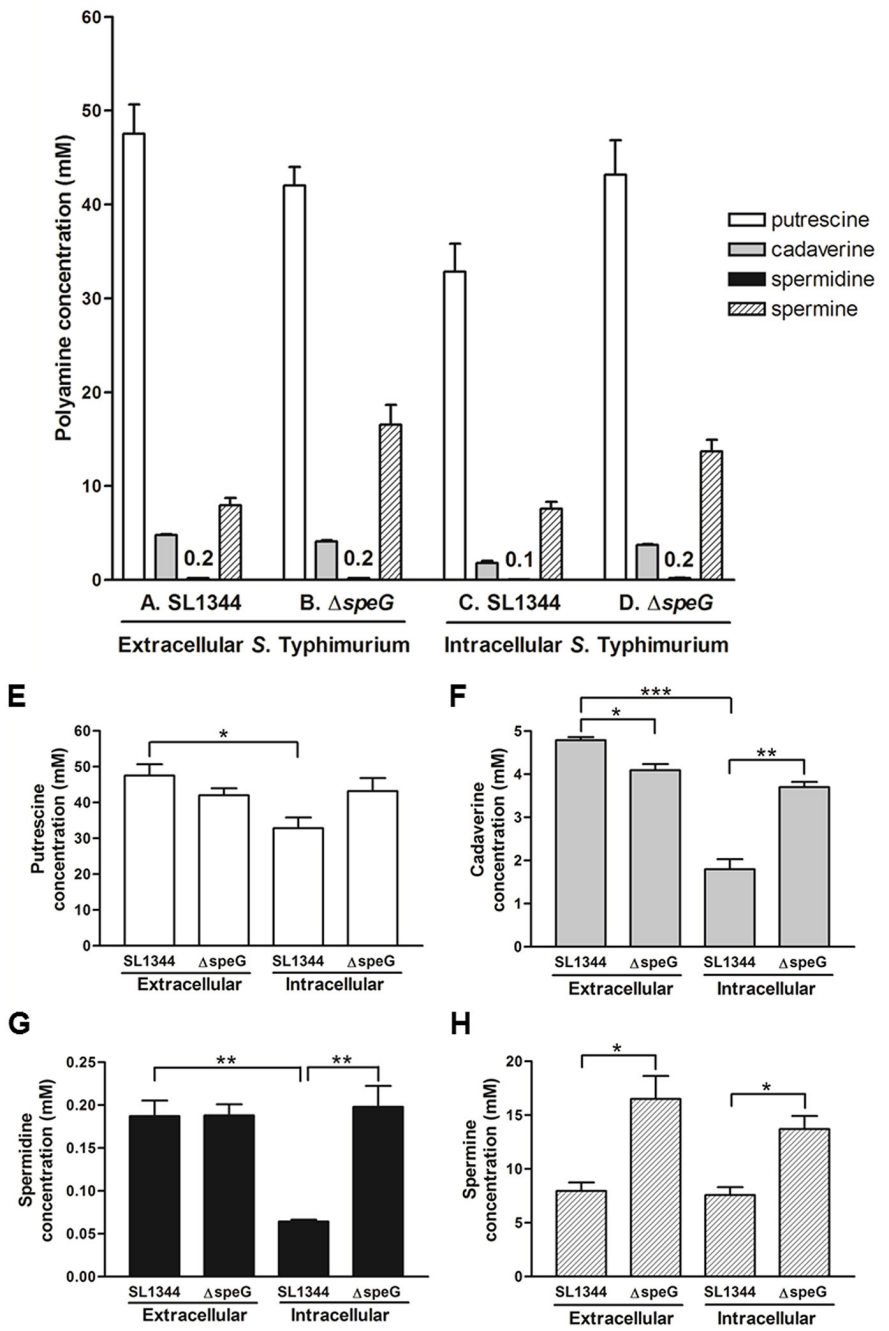
HPLC quantification of cellular polyamines in *S*. Typhimurium SL1344 and Δ*speG* before and after invasion in Caco-2 cells for 18 h. By using the same protocol as that for obtaining output pool B in the intracellular bacterial replication assay, the cellular polyamines of *S*. Typhimurium SL1344 and Δ*speG* before **(A,B)** and after **(C,D)** 18-h intracellular internalization in Caco-2 cells were extracted through TCA precipitation and quantified through HPLC for measuring the four major polyamines. The concentration of each polyamine in *S*. Typhimurium was compared between the SL1344 and Δ*speG* strains extracellularly and intracellularly **(E–H)**. For the same strain of SL1344 or Δ*speG*, the concentration of each polyamine was also compared between extracellular bacteria from mid-log cultures and intracellular bacteria after invasion in Caco-2 cells. Statistical significances in the comparisons are indicated by asterisks (^*^p < 0.05, ^**^p < 0.01, and ^***^p < 0.001; *n* = 3).

To quantify the concentrations of putrescine, cadaverine, spermidine, and spermine in *S*. Typhimurium SL1344 before and after invasion in Caco-2 cells and to investigate whether *speG* affects their concentrations in *S*. Typhimurium SL1344 outside and inside Caco-2 cells, as well as extracellular and intracellular *S*. Typhimurium SL1344, we performed HPLC to quantify these polyamines from the mid-log cultures of both strains and from their 18-h postinfection intracellular concentrations in Caco-2 cells. Our HPLC analysis of the mid-log cultures of *S*. Typhimurium SL1344 revealed that the concentration of putrescine (47.5 mM), the predominant polyamine, was approximately six times higher than that of spermine (8.0 mM) and approximately 10 times higher than that of cadaverine (4.8 mM). The concentration of spermidine (0.2 mM) was the lowest (Figure 2A). The order of the cellular contents of all these cytoplasmic polyamines was the same in *S*. Typhimurium after the deletion of *speG* (Figure 2B), as well as in intracellular *S*. Typhimurium and Δ*speG* (Figures 2C,D). In summary, putrescine, spermine, and cadaverine were the polyamines with the highest concentrations in extracellular and intracellular *S*. Typhimurium SL1344 and Δ*speG* (Figures 2A–D).

### *speG* suppresses the concentration of spermine but increases that of cadaverine in extracellular *S*. typhimurium

To investigate the effects of *speG* on the contents of polyamines in *S*. Typhimurium, the HPLC-quantified concentrations of the polyamines in *S*. Typhimurium SL1344 and Δ*speG* were compared. *S*. Typhimurium Δ*speG* contained significantly lower concentrations of cadaverine and higher concentrations of spermine than did *S*. Typhimurium SL1344 (4.1 vs. 4.8 mM and 16.5 vs. 8.0 mM, respectively; Figures 2F,H), indicating that *speG* expression might enhance cadaverine production and suppress spermine synthesis. The depletion of *speG* did not significantly change the content of spermidine in *S*. Typhimurium.

### Biosynthesis of putrescine, cadaverine, and spermidine is reduced in intracellular *S*. typhimurium in Caco-2 cells and *speG* is involved in inhibition of polyamine production in intracellular *S*. typhimurium

After bacterial invasion in Caco-2 cells for 18 h, the concentrations of putrescine, cadaverine, and spermidine significantly decreased in *S*. Typhimurium SL1344 (32.8 vs. 47.5 mM, 1.8 vs. 4.8 mM, and 0.2 vs. 0.1 mM, respectively; Figures 2E–G); however, the levels of spermine were similar to those in extracellular *S*. Typhimurium SL1344 (7.6 vs. 8 mM; Figure 2H). Briefly, the biosynthesis of purescine, cadaverine, and spermidine was significantly suppressed in *S*. Typhimurium SL1344 in Caco-2 cells 18 h after invasion. To verify the hypothesis that deficiency in the intracellular replication of *S*. Typhimurium Δ*speG* is due to its quantitative alteration in polyamines, the concentrations of four polyamines were compared between intracellular *S*. Typhimurium SL1344 and Δ*speG*. We observed that the concentrations of putrescine, spermine, and cadaverine were higher in intracellular *S*. Typhimurium Δ*speG* than in intracellular *S*. Typhimurium SL1344 (Figures 2C,D). Briefly, the deletion of *speG* significantly increased the concentrations of cadaverine, spermidine, and spermine in intracellular *S*. Typhimurium in Caco-2 cells for 18 h (3.7 vs. 1.8 mM, 0.2 vs. 0.1 mM, and 13.7 vs. 7.6 mM, respectively; Figures 2F–H) and non-significantly increased the concentration of putrescine (43.2 vs. 32.8 mM, *p* = 0.093; Figures 2E). These results suggested that *speG* can inhibit the biosynthesis of polyamines in intracellular *S*. Typhimurium, with a different tendency for modulating putrescine, cadaverine, and spermidine compared with extracellular *S*. Typhimurium.

### Intracellular proliferation of *S*. typhimurium is dose-dependently suppressed by putrescine, cadaverine, and spermine

**Figure 3:**
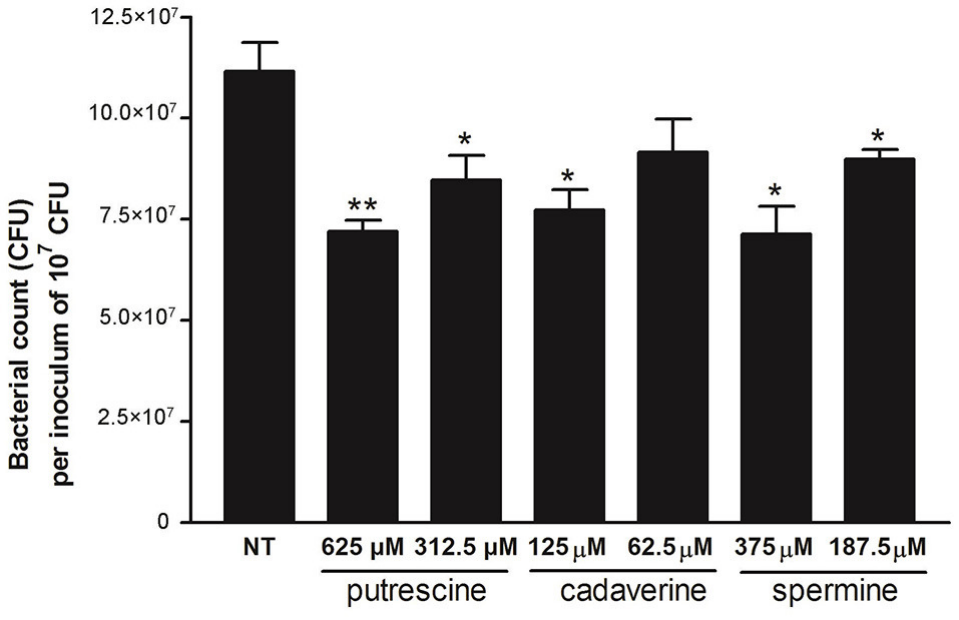
Polyamine suppression assays of putrescine, cadaverine, and spermine in intracellular replication of *S*. Typhimurium SL1344 in Caco-2 cells. By using the same protocol as that for obtaining output pool B of the intracellular bacterial replication assay, confluent Caco-2 cells in 12-well plates were infected with overnight cultures of *S*. Typhimurium SL1344, and the infected cells were treated with putrescine, spermine, and cadaverine during the last 15 h in concentrations estimated from the HPLC quantification analysis. Statistical significances in the intracellular bacterial concentrations of *S*. Typhimurium SL1344 between the treated and untreated groups are indicated by asterisks (^*^p < 0.05 and ^**^p < 0.01; *n* = 3).

Because non-replicating intracellular *S*. Typhimurium Δ*speG* contains higher concentrations of putrescine, spermine, and cadaverine, we hypothesized that the accumulation of these polyamines inhibits the intracellular replication of *S*. Typhimurium SL1344. A polyamine suppression assay similar to the 18-h intracellular replication assay was performed by infecting Caco-2 cells with *S*. Typhimurium SL1344 for 2 h, followed by 1-h treatment with gentamicin to kill extracellular bacteria and 15-h coincubation of Caco-2 cells with intracellular *S*. Typhimurium SL1344 and the three polyamines in two estimated concentrations. The HPLC analysis of polyamines in intracellular *S*. Typhimurium Δ*speG* was conducted after 2-h amplification through the shaking incubation of bacteria released from lysed Caco-2 cells, and the doubling time of *S*. Typhimurium was 20–30 min. Therefore, the estimated concentrations of putrescine (625 and 312 μM), cadaverine (125 and 62.5 μM), and spermine (375 and 187.5 μM) were 2^5^ to 2^6^ times diluted compared with their corresponding concentrations (43.2, 3.7, and 13.7 mM) in the HPLC quantification (intracellular *S*. Typhimurium Δ*speG*; Figure 2D). Intracellular *S*. Typhimurium SL1344 (1.1 × 10^8^ CFU per inoculum of 10^7^ CFU) was significantly suppressed by 625 and 312.5 μM of putrescine (7.2 × 10^7^ CFU per inoculum of 10^7^ CFU, *p* = 0.006, and 8.5 × 10^7^ CFU per inoculum of 10^7^ CFU, *p* = 0.04, respectively), 125 μM of cadaverine (7.7 × 10^7^ CFU per inoculum of 10^7^ CFU, *p* = 0.017), and 375 and 187.5 μM of spermine (7.1 × 10^7^ CFU per inoculum of 10^7^ CFU, *p* = 0.015 and 9 × 10^7^ CFU per inoculum of 10^7^ CFU, *p* = 0.04, respectively). The concentration of intracellular *S*. Typhimurium SL1344 was non-significantly lower in Caco-2 cells treated with 62.5 μM of cadaverine (9.2 × 10^7^ CFU per inoculum of 10^7^ CFU, *p* = 0.138) than that of Caco-2 cells not treated with polyamines (Figure 3). Altogether, *S*. Typhimurium SL1344 was dose-dependently suppressed by putrescine, cadaverine, and spermine (Figure 3).

### *speG* is involved in suppressing upregulation of genes associated with periplasmic nitrate reductase system, glucarate metabolism, phosphotransferase system, cytochromes, and succinate reductase complex

To investigate the effects of *speG* on the expression of other genes in *S*. Typhimurium, the entire RNA transcriptome of *S*. Typhimurium Δ*speG* was compared with that of *S*. Typhimurium SL1344 (Data Sheet 1). In the mid-log cultures, 29 genes were significantly upregulated in *S*. Typhimurium Δ*speG* compared with *S*. Typhimurium SL1344, including *yojF* and *napF*, which are involved in the periplasmic nitrate reductase system; *ygcX, ygcZ, garL, garR*, and SL1344_2942, which are associated with glucarate metabolism; SL1344_3736 and SL1344_4467, which are related to the phosphotransferase system; *cyoA, cyoB*, and *cyoC*, which encode cytochrome-related proteins; and *sdhA, sdhB, sdhC*, and *sdhD*, which are involved in the succinate reductase complex (Table 2A). Moreover, *narK, malK, ybfM*, SL1344_2997, SL1344_3662, *dctA, hutU*, and *hutH* as well as five genes encoding hypothetical proteins were significantly upregulated (Table 2A). Thus, *speG* could be involved in suppression of the expression of these significantly upregulated genes in *S*. Typhimurium.

**Table 2:** The 29 significantly upregulated genes **(A)** and the 110 significantly downregulated genes **(B)** of the *S*. Typhimurium Δ*speG* mutant compared with its parental wild-type strain SL1344.

**Gene name**	**Target ID**	**Function**	**Log2 fold change**
**(A) UPREGULATED GENES**			
**Genes of Periplasmic Nitrate Reductase System**			
*yojF*	SL1344_2230	Putative napAB assembly protein	3.782
*napF*	SL1344_2231	Ferredoxin-type protein NapF	3.073
**Genes of Glucarate Metabolism**			
*ygcZ*	SL1344_2943	Glucarate transporter	3.248
*garL*	SL1344_3222	5-Keto-4-deoxy-D-glucarate aldolase	2.819
*garR*	SL1344_3221	2-Hydroxy-3-oxopropionate reductase	2.760
*ygcX*	SL1344_2941	Glucarate dehydratase	2.037
SL1344_2942	SL1344_2942	Glucarate dehydratase	1.257
**Genes of Phosphotransferase System**			
SL1344_3736	SL1344_3736	Putative PTS system protein	1.673
SL1344_4467	SL1344_4467	PTS transport system, IIB component	1.621
**Genes of Cytochromes**			
*cyoA*	SL1344_0437	Cytochrome o ubiquinol oxidase subunit II	1.668
*cyoB*	SL1344_0436	Cytochrome o ubiquinol oxidase subunit I	1.406
*cyoC*	SL1344_0716	Cytochrome o ubiquinol oxidase subunit III	1.227
**Genes of Succinate Dehydrogenase Complex**			
*sdhD*	SL1344_0715	Succinate dehydrogenase hydrophobic membrane anchor protein	1.239
*sdhA*	SL1344_0435	Succinate dehydrogenase flavoprotein subunit	1.121
*sdhB*	SL1344_0717	Succinate dehydrogenase iron-sulfur protein	1.120
*sdhC*	SL1344_0714	Succinate dehydrogenase cytochrome b-556 subunit	1.027
**Other Genes**			
*narK*	SL1344_1693	Nitrite extrusion protein	4.443
*malK*	SL1344_4167	Maltose–maltodextrin transport ATP-binding protein	3.343
*ybf M*	SL1344_0669	Putative outer membrane protein	2.372
SL1344_2997	SL1344_2997	Acetyl-CoA acetyltransferase	2.089
SL1344_3662	SL1344_3662	Putative racemase	1.552
SL1344_0211	SL1344_0211	Hypothetical protein	1.495
*dctA*	SL1344_3579	C4-dicarboxylate transport protein	1.454
SL1344_2940	SL1344_2940	Hypothetical protein	1.319
*hutU*	SL1344_0767	Urocanate hydratase	1.237
SL1344_3732	SL1344_3732	Hypothetical protein	1.227
SL1344_0790	SL1344_0790	Hypothetical protein	1.163
SL1344_1227	SL1344_1227	Hypothetical protein	1.010
*hutH*	SL1344_0768	Histidine ammonia-lyase	1.002
**(B) DOWNREGULATED GENES**			
**Flagellar Genes**			
*flhA*	SL1344_1848	Flagellar biosynthetic protein FlhA	−1.994
*flhB*	SL1344_1849	Flagellar biosynthetic protein FlhB	−1.918
*fliP*	SL1344_1908	Flagellar biosynthetic protein FliP	−1.776
*fliB*	SL1344_1887	Flagellin lysine-N-methylase	−1.751
*fliQ*	SL1344_1909	Flagellar biosynthetic protein FliQ	−1.688
*flgI*	SL1344_1118	Flagellar P-ring protein	−1.648
*fliN*	SL1344_1906	Flagellar motor switch protein FliN	−1.645
*fliO*	SL1344_1907	Flagellar biosynthesis protein	−1.639
*fliF*	SL1344_1898	Flagellar basal-body M-ring protein	−1.633
*flhE*	SL1344_1847	Flagellar protein FlhE	−1.603
*fliR*	SL1344_1910	Flagellar biosynthetic protein FliR	−1.601
*flgH*	SL1344_1117	Flagellar L-ring protein	−1.586
*flgL*	SL1344_1121	Flagellar hook-associated protein 3	−1.516
*flgJ*	SL1344_1119	Flagellar protein FlgJ	−1.507
*fliM*	SL1344_1905	Flagellar motor switch protein FliM	−1.473
*fliA*	SL1344_1885	RNA polymerase sigma transcription factor for flagellar operon	−1.458
*fliL*	SL1344_1904	Flagellar biosynthesis protein	−1.415
*fliJ*	SL1344_1902	Flagellar biosynthesis protein	−1.401
*fliK*	SL1344_1903	Flagellar hook-length control protein	−1.361
*fliH*	SL1344_1900	Flagellar assembly protein FliH	−1.339
*fliG*	SL1344_1899	Flagellar motor switch protein FliG	−1.316
*fliD*	SL1344_1889S	Flagellar hook associated protein FliD	−1.251
*fliS*	L1344_1890	Flagellar protein FliS	−1.211
*fliT*	SL1344_1891	Flagellar protein FliT	−1.056
**Fimbrial Genes**			
*fimW*	SL1344_0545	Fimbriae w protein	−2.084
*fimC*	SL1344_0538	Fimbrial chaperone protein	−1.694
*fimD*	SL1344_0539	Outer membrane usher protein FimD	−1.623
*fimI*	SL1344_0537	Major pilin protein	−1.442
*stjC*	SL1344_4500	Fimbrial chaperone	−1.386
**T3SS-Related Genes**			
*sopD*	SL1344_2924	Hypothetical protein	−3.273
*sopA*	SL1344_2043	Secreted protein SopA	−3.273
*sopE2*	SL1344_1784	Invasion-associated secreted effector protein (sopE2)	−2.996
*slrP*	SL1344_0776	Leucine rich repeat	−2.537
*spiR*	SL1344_1326	Putative two-component sensor kinase	−1.974
*ssrB*	SL1344_1325	Putative two-component response regulator	−1.705
*srfC*	SL1344_1526	Putative virulence effector protein	−1.506
*srfA*	SL1344_1524	Putative virulence effector protein	−1.451
*srfB*	SL1344_1525	Putative virulence effector protein	−1.361
*ssaI*	SL1344_1342	Putative pathogenicity island protein	−1.324
*ssaJ*	SL1344_1343	Putative pathogenicity island lipoprotein	−1.230
*sseA*	SL1344_1331	T3SS chaperone	−1.222
*ssaH*	SL1344_1341	Type three secretion system apparatus	−1.218
*sseB*	SL1344_1332	Putative pathogenicity island effector protein	−1.129
*ssaG*	SL1344_1340	Putative pathogenicity island protein	−1.019
**Other Genes**			
*speG*	SL1344_1432	Spermidine N1-acetyltransferase	−8.818
*asnA*	SL1344_3844	Asparagine synthetase A	−3.380
SL1344_3163	SL1344_3163	Hypothetical protein	−3.327
*pipB*	SL1344_1027	Hypothetical protein	−3.148
*siiE*	SL1344_4197	Hypothetical protein	−3.113
*pipC*	SL1344_1029	Cell invasion protein	−2.944
SL1344_1265	SL1344_1265	Putative DNA/RNA non-specific endonuclease	−2.843
*siiF*	SL1344_4198	Putative type-1 secretion protein	−2.823
SL1344_0337	SL1344_0337	Hypothetical protein	−2.473
SL1344_1028	SL1344_1028	Inner membrane protein	−2.278
*ugtL*	SL1344_1531	Hypothetical protein	−2.168
SL1344_1846	SL1344_1846	Hypothetical protein	−2.072
*pagD*	SL1344_1183	Putative outer membrane virulence protein (PagD)	−2.006
SL1344_1330A	SL1344_1330A	Hypothetical protein	−1.957
SL1344_1795	SL1344_1795	Hypothetical protein	−1.932
SL1344_4434	SL1344_4434	Hypothetical protein	−1.929
*baeS*	SL1344_2107	Putative two-component system sensor kinase	−1.907
*pagC*	SL1344_1184	Outer membrane invasion protein (PagC)	−1.863
SL1344_2763	SL1344_2763	Hypothetical protein	−1.827
SL1344_1083	SL1344_1083	Hypothetical protein	−1.765
SL1344_4435	SL1344_4435	Hypothetical protein	−1.721
SL1344_1794	SL1344_1794	Putative inner membrane protein	−1.705
SL1344_1530A	SL1344_1530A	Hypothetical protein	−1.680
SL1344_1783	SL1344_1783	Hypothetical protein	−1.666
SL1344_1867	SL1344_1867	Putative lipoprotein	−1.654
*gtgE*	SL1344_0995	Bacteriophage encoded virulence factor	−1.626
SL1344_0532	SL1344_0532	Hypothetical protein	−1.615
*pagO*	SL1344_1793	Inner membrane protein (PagO)	−1.606
*Iap*	SL1344_2915	Alkaline phosphatase isozyme conversion protein	−1.604
SL1344_0544	SL1344_0544	Hypothetical protein	−1.597
SL1344_0973	SL1344_0973	Bacteriophage protein	−1.467
*yegB*	SL1344_2106	Putative transporter protein	−1.428
SL1344_2984	SL1344_2984	Hypothetical protein	−1.425
SL1344_0947	SL1344_0947	Bacteriophage protein	−1.421
SL1344_0974	SL1344_0974	Bacteriophage protein	−1.415
SL1344_1345	SL1344_1345	Putative pathogenicity island protein	−1.389
*hyaD2*	SL1344_1465	Hydrogenase 1 maturation protease	−1.376
SL1344_0948	SL1344_0948	Bacteriophage protein	−1.361
*hyaF2*	SL1344_1462	Hydrogenase-1 operon protein HyaF2	−1.341
SL1344_0531	SL1344_0531	Putative exported outer membrane protein	−1.337
SL1344_1085	SL1344_1085	Hypothetical protein	−1.336
*cheA*	SL1344_1856	Chemotaxis protein CheA	−1.317
*asnB*	SL1344_0662	Asparagine synthetase B	−1.314
SL1344_0739	SL1344_0739	Putative hydrolyase	−1.305
SL1344_1188	SL1344_1188	Hypothetical protein	−1.286
SL1344_0975	SL1344_0975	Bacteriophage protein	−1.269
*phoN*	SL1344_4255	Non-specific acid phosphatase	−1.248
*C1*	SL1344_0951	Transcriptional activator-regulatory protein	−1.238
*ybcI*	SL1344_0533	Hypothetical protein	−1.210
*ecnR*	SL1344_4274	Transcriptional regulator	−1.204
SL1344_1928	SL1344_1928	Putative bacteriophage protein	−1.193
*gpO*	SL1344_2591	Bacteriophage replication protein	−1.136
SL1344_3128	SL1344_3128	Hypothetical protein	−1.111
SL1344_0993	SL1344_0993	Bacteriophage protein	−1.107
SL1344_1464	SL1344_1464	Hydrogenase isoenzymes formation protein	−1.103
*yfeA*	SL1344_2378	Hypothetical protein	−1.103
*pphA*	SL1344_1782	Serine–threonine protein phosphatase	−1.101
*yjcC*	SL1344_4200	Hypothetical protein	−1.074
SL1344_1628	SL1344_1628	Hypothetical protein	−1.049
SL1344_3130	SL1344_3130	Hypothetical protein	−1.034
SL1344_1461	SL1344_1461	Putative ATP/GTP-binding protein	−1.029
*pgtE*	SL1344_2363	Outer membrane protease E	−1.024
SL1344_1344	SL1344_1344	Putative pathogenicity island protein	−1.013
*hyaE2*	SL1344_1463	Hydrogenase-1 operon protein HyaE2	−1.006
*rtcB*	SL1344_3486	Hypothetical protein	−1.005
SL1344_1845	SL1344_1845	Penicillin-binding protein	−1.002

### *speG* is involved in flagellar biosynthesis, fimbrial expression, and T3SS and T1SS functions

In the mid-log cultures, 110 genes were significantly downregulated in *S*. Typhimurium Δ*speG* compared with *S*. Typhimurium SL1344 in the RNA microarrays (Table 2B). First, *speG* was the most significantly downregulated gene (−8.818 log2 fold change) in *S*. Typhimurium Δ*speG*, validating the successful deletion of *speG* in its parental wild-type *S*. Typhimurium SL1344 (Table 2B). However, *speG* deletion did not significantly affect the expression of genes involved in polyamine metabolism, namely *speA* (−0.52 log2 fold change, *p* = 0.44), *speB* (−0.07 log2 fold change, *p* = 0.91), *speC* (0.99 log2 fold change, *p* = 0.22), *speD* (−0.28 log2 fold change, *p* = 0.70), *speE* (−0.21 log2 fold change, *p* = 0.88), *cadA* (−0.99 log2 fold change, *p* = 0.46), and *metK* (−0.51 log2 fold change, *p* = 0.76).

**Figure 4:**
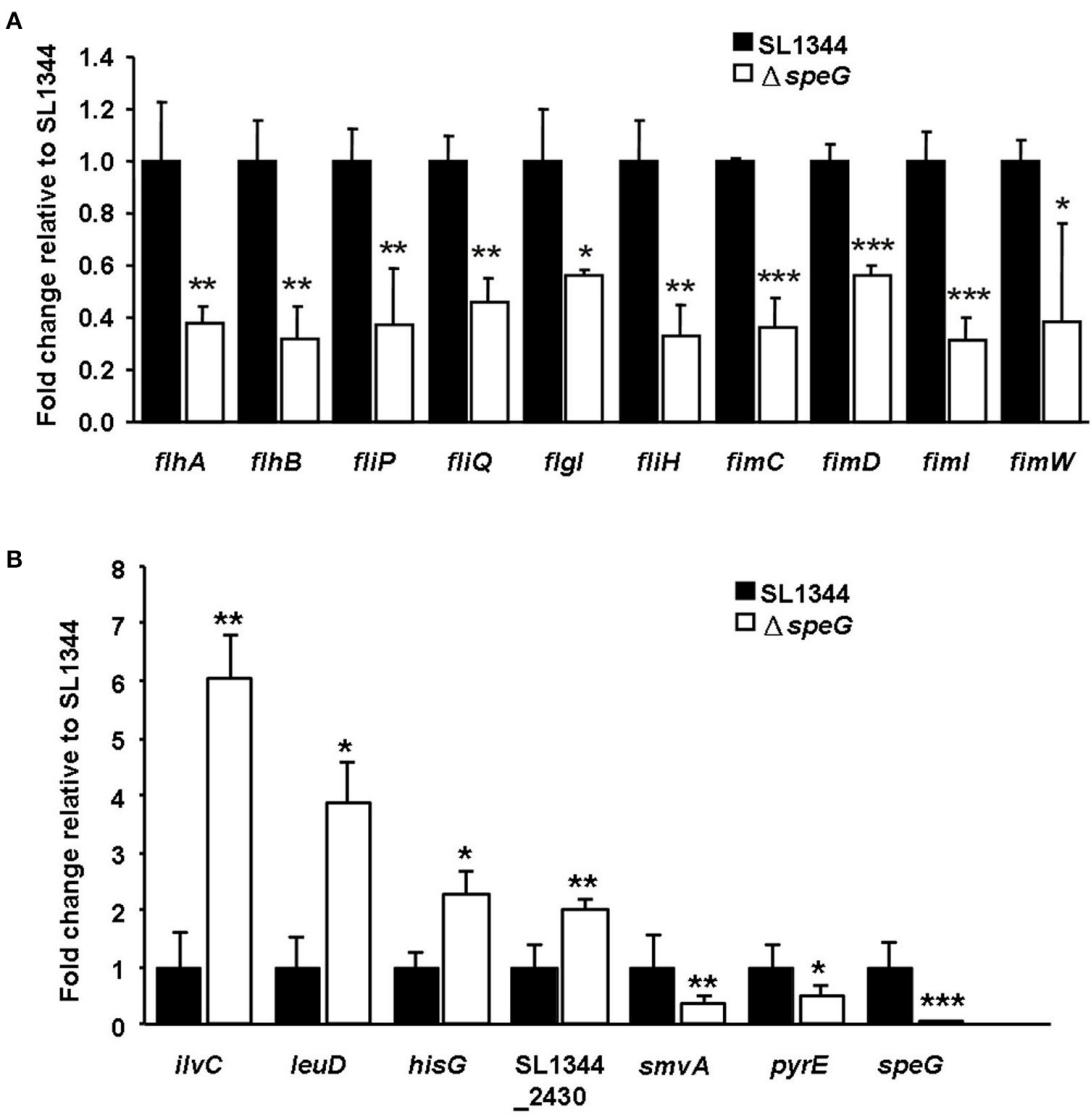
Quantitative real-time polymerase chain reaction analysis of 17 selected genes for validation of the RNA microarray data. **(A)** The mRNA expression levels of the six flagellar genes and four fimbrial genes were significantly downregulated in *S*. Typhimurium Δ*speG* relative to its parental wild-type strain SL1344. **(B)** The mRNA expressions of *ilvC, leuD, hisG*, and SL1344_2430 were significantly upregulated and those of *smvA, pyrE*, and *speG* were significantly downregulated in intracellular *S*. Typhimurium Δ*speG* compared with *S*. Typhimurium SL1344 after invasion in Caco-2 cells for 18 h. Statistical significances in mRNA expression of the selected genes between *S*. Typhimurium SL1344 and Δ*speG* are indicated by asterisks (^*^p < 0.05, ^**^p < 0.01, ^***^p < 0.001; *n* = 3).

The transcriptome of the *S*. Typhimurium Δ*speG* mutant relative to its parental wild-type strain SL1344 revealed that the protein functions of the significantly downregulated genes include flagellar biosynthesis, fimbrial chaperone, pathogenicity island 4 (e.g., *siiE*), and type I secretion system (e.g., *siiF*; Table 2B), suggesting the involvement of *speG* in the flagellar motility regulation of *S*. Typhimurium. Moreover, many T3SS-related genes were significantly downregulated after *speG* deletion in *S*. Typhimurium SL1344 (Table 2B). These RNA microarray results were confirmed through qRT-PCR, which demonstrated significantly downregulated mRNA expression of six flagellar genes (*flhA, flhB, fliP, fliQ, flgI*, and *fliH*) and four fimbrial genes (*fimC, fimD, fimI*, and *fimW*; Figure 4A).

### *speG* is involved in suppressing upregulation of genes in the *ilv*–*leu* operon and histidine operon of intracellular *S*. typhimurium after invasion in Caco-2 cells

To understand whether *speG* affects the gene expression of intracellular *S*. Typhimurium, the transcriptomes of intracellular *S*. Typhimurium SL1344 and its Δ*speG* mutant were compared after internalization in Caco-2 cells for 18 h. Compared with the expression pattern in intracellular *S*. Typhimurium SL1344, 1,964 genes were upregulated and 2,664 genes were downregulated in the intracellular *S*. Typhimurium Δ*speG* mutant (Data Sheet 2). The depletion of *speG* led to the significant upregulation of 11 genes in intracellular *S*. Typhimurium, namely *ilvC, ilvM, ilvG, ilvA, leuD, leuC*, and *leuB* in the *ilv*–*leu* operon and *hisG* and *hisC* in the histidine operon (Table 3A). Only four genes, namely *speG, smvA, pyre*, and *citD2*, were significantly downregulated in the intracellular *S*. Typhimurium Δ*speG* mutant (Table 3B). The RNA microarray results were validated through qRT-PCR, which revealed the significantly upregulated mRNA expression of four genes (*ilvC, leuD, hisG*, and SL1344_2430) and significantly downregulated mRNA expression of three genes (*smvA, pyrE*, and *speG*; Figure 4B).

**Table 3:** The 11 significantly upregulated genes **(A)** and the four significantly downregulated genes **(B)** of the intracellular *S*. Typhimurium Δ*speG* mutant compared with its parental wild-type strain SL1344 within Caco-2 cells.

**Gene**	**Target ID**	**Function**	**Log2 fold change**
**(A) SIGNIFICANTLY UPREGULATED GENES**			
*ilvC*	SL1344_3869	Ketol-acid reductoisomerase	1.733
*leuD*	SL1344_0110	3-Isopropylmalate dehydratase small subunit	1.694
*leuC*	SL1344_0111	3-Isopropylmalate dehydratase large subunit	1.670
*hisG*	SL1344_2048	ATP phosphoribosyltransferase	1.499
*ilvM*	SL1344_3862	Acetohydroxy acid synthase II, small subunit	1.351
*leuB*	SL1344_0112	3-Isopropylmalate dehydrogenase	1.333
*ilvG*	SL1344_3861	Acetolactate synthase large subunit	1.141
*ilvA*	SL1344_3865	Threonine deaminase	1.077
SL1344_2430	SL1344_2430	Putative cobalamin adenosyltransferase	1.052
SL1344_1416	SL1344_1416	Putative membrane transport protein	1.045
*hisC*	SL1344_2050	Histidinol-phosphate aminotransferase (imidazole)	1.029
**(B) SIGNIFICANTLY DOWNREGULATED GENES**			
*speG*	SL1344_1432	Spermidine N1-acetyltransferase	−6.578
*smvA*	SL1344_1505	Methyl viologen resistance protein SmvA	−2.885
*pyrE*	SL1344_3699	Orotate phosphoribosyltransferase	−1.207
*citD2*	SL1344_0060	Citrate lyase acyl carrier protein	−1.043

### *speG* affects flagellation and motility of *S*. typhimurium and exogenous polyamins attenuate motility of *S*. typhimurium

**Figure 5:**
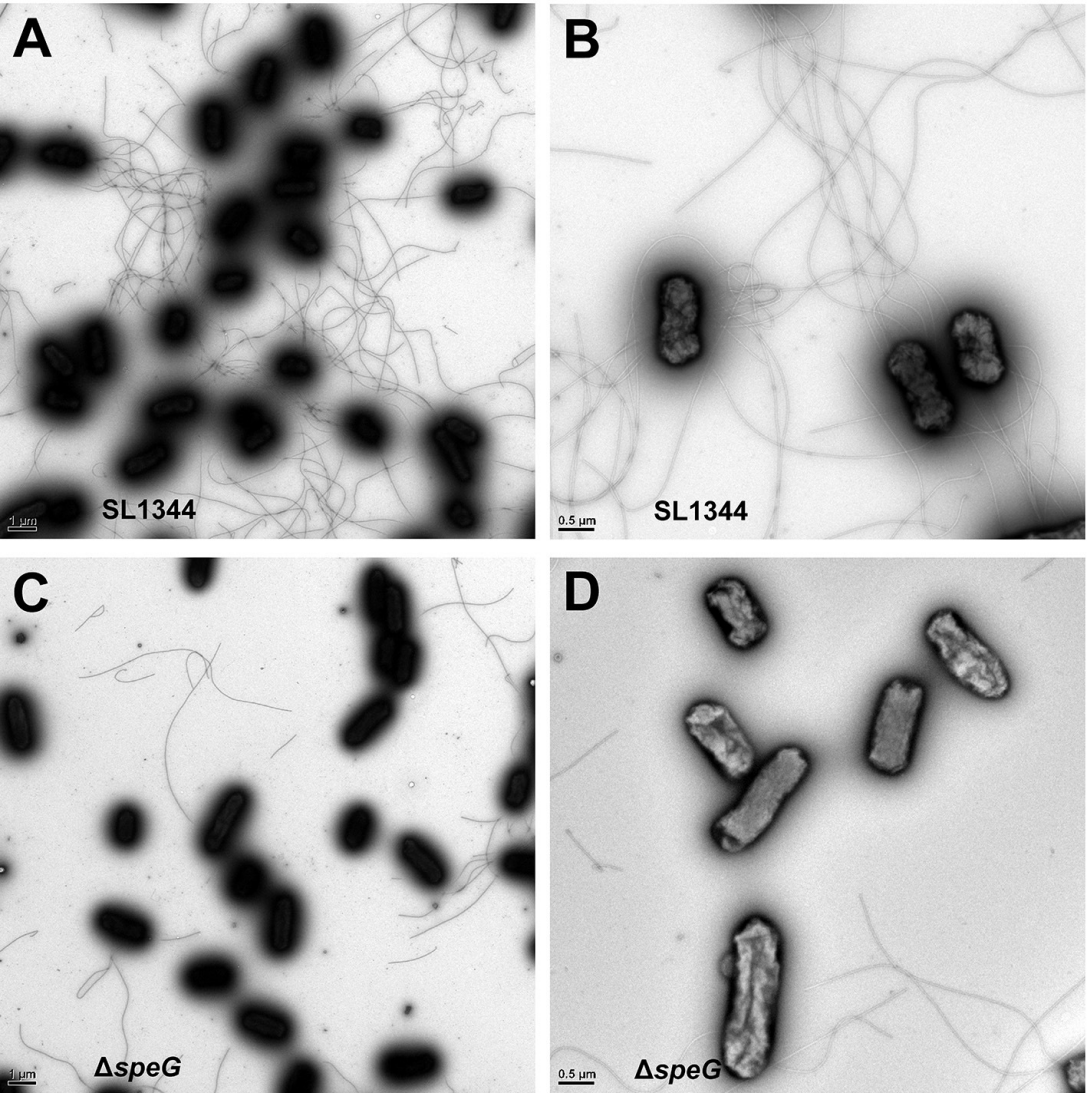
Transmission electron micrographs of *S*. Typhimurium SL1344 and Δ*speG*. Transmission electron micrographs after negative staining revealed the morphology of **(A,B)** *S*. Typhimurium SL1344 and **(C,D)** Δ*speG*. Numerous long flagella were observed in *S*. Typhimurium SL1344 [magnification: **(A)** 10,000× and **(B)** 22,500×]. Only a small number of fragmented flagella were observed in *S*. Typhimurium Δ*speG* [magnification: **(C)** 10,000× and **(D)** 22,500×].

**Figure 6:**
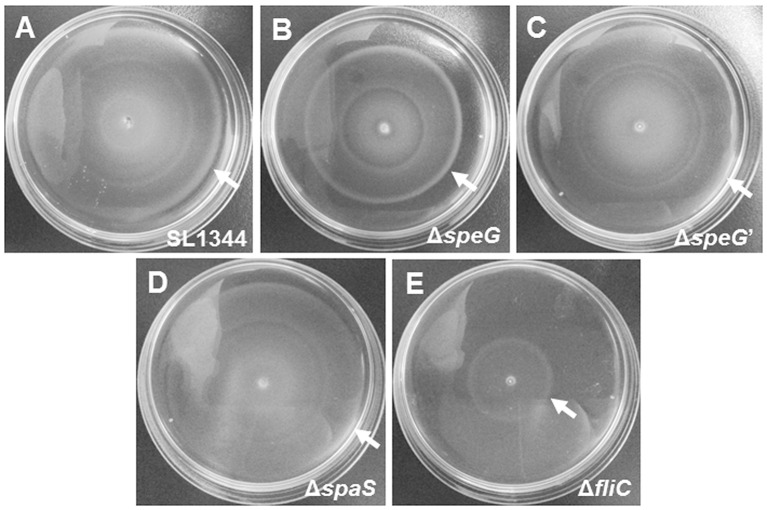
Bacterial motility assays of *S*. Typhimurium SL1344 and Δ*speG*. The motilities of *S*. Typhimurium SL1344 **(A)**, Δ*speG* **(B)**, Δ*speG*′ **(C)**, Δ*spaS* **(D)**, and Δ*fliC* **(E)** were examined by bacterial inoculation on semisolid agar plates with 6-h incubation at 37°C. *S*. Typhimurium SL1344, Δ*speG*′, and Δ*spaS* similarly exhibited the maximal diameters of their motility zones. The motility zone of Δ*speG* was smaller than that of the aforementioned strains, but still larger than that of Δ*fliC*.

**Figure 7:**
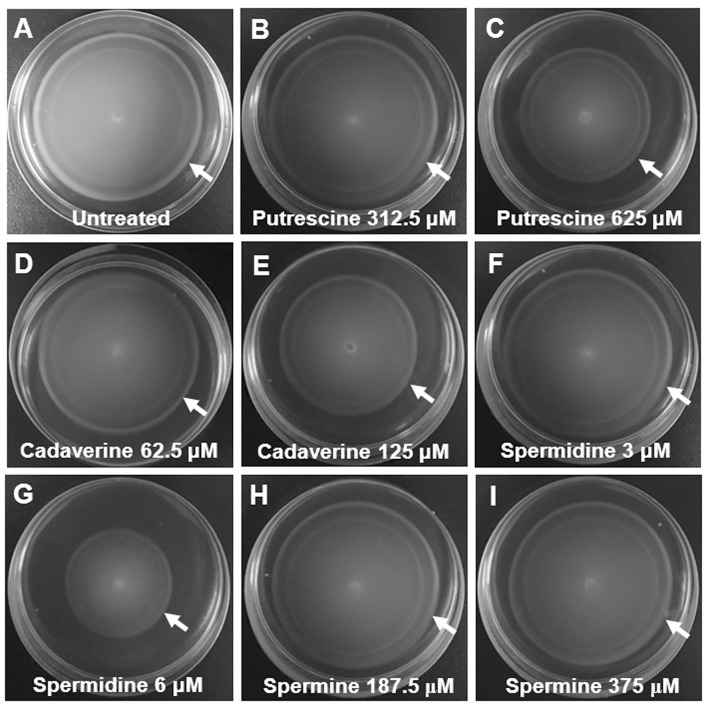
Polyamine-suppressing bacterial motility assays of *S*. Typhimurium SL1344. The motilities of *S*. Typhimurium SL1344 were examined by bacterial inoculation on semisolid agar plates supplemented with four polyamines after 6-h incubation at 37°C. The motility zones of *S*. Typhimurium SL1344 in the semisolid LB agar plates supplemented with putrescine (625 μM; **C**), cadaverine (125 μM; **E**), and spermidine (6 μM; **G**) were smaller than that of *S*. Typhimurium SL1344 in the plates containing no polyamine **(A)**. The motility zones of *S*. Typhimurium SL1344 were slightly inhibited by cadaverine (62.5 μM; **D**), but not suppressed by putrescine (312.5 μM; **B**), spermidine (3 μM; **F**), and two concentrations of spermine (187.5 μM; **H** and 375 μM; **I**) in the semisolid LB agar plates.

To validate the RNA microarray data on the involvement of *speG* in flagellar biosynthesis, the bacterial morphology and motility of *S*. Typhimurium SL1344 and Δ*speG* were examined. Transmission electron micrographs after negative staining revealed sparse defective flagella in *S*. Typhimurium Δ*speG* compared with the wild-type SL1344 strain (Figure 5). Moreover, the motility of *S*. Typhimurium Δ*speG* was poorer than that of the wild-type SL1344 strain but better than that of *S*. Typhimurium Δ*fliC* (Figure 6). The bacterial motility was restored after *speG* complementation in the Δ*speG* mutant (Figure 6C), suggesting the influence of *speG* on bacterial motility. In addition, the motility of S. Typhimurium SL1344 was dose-dependently attenuated by exogenous putrescine, cadaverine, and spermidine (Figure 7).

## Discussion

According to our review of relevant literature, the present study is the first to demonstrate that *speG* is required for the intracellular replication of *Salmonella* in human non-phagocytic cells. Our results revealed that the effect of *speG* on the intracellular replication of *S*. Typhimurium in HeLa cells is time dependent (Figure 1A). Moreover, we observed that *speG* influenced the intracellular replication of *S*. Typhimurium in four cell lines as hosts (Figures 1B–E). Although the *speG* mutant was attenuated in bacterial invasion in Caco-2 cells, it was more significantly attenuated in bacterial replication after 18-h internalization in Caco-2 cells (Figure 1C), suggesting the effect of *speG* on the suppression of intracellular bacterial replication. The depletion of *speG* did not affect the internalization of *S*. Typhimurium in THP-1 cells (Figure 1E), which indicated that the *speG* phenotype is mainly exhibited within host cells rather than outside the host cells. In *E. coli, speG* encodes SAT, which catalyzes spermidine to N^1^- or N^8^-acetylspermidine (Igarashi and Kashiwagi, 2010; Barbagallo et al., 2011; Jelsbak et al., 2012), which can be secreted by the bacteria to inactivate the toxicity of excessive spermidine (Fukuchi et al., 1995) and can reduce intracellular polyamine levels to prevent spermidine toxicity under stressful growth conditions (Limsuwun and Jones, 2000). The amino acid sequence of SAT in *E. coli* is similar to that in *Enterococcus faecalis, Pseudomonas aeruginosa* plasmid pSCH884, *Klebsiella pneumoniae, Serratia marcessencens*, and *Agrobacterium tumefaciences* (Fukuchi et al., 1994). However, *speG* is not present in all bacteria and is lost in *Shigella*, resulting in higher concentrations of endogenous spermidine (Barbagallo et al., 2011). Although the absence of *speG* enhances the survival of *Shigella* to oxidative stress and the adverse environments inside macrophages (Barbagallo et al., 2011), our study demonstrated the requirement of *speG* for the intracellular replication of *S*. Typhimurium in human macrophages and epithelial cells. *speG* deletion did not affect the ability of *Shigella flexneri* to infect HeLa cells (Barbagallo et al., 2011), but it caused spermidine accumulation to reduce the intracellular viability of *E. coli* (Fukuchi et al., 1995). These observations are consistent with our results that the loss of *speG* in *S*. Typhimurium did not impair bacterial invasion in HeLa cells but attenuated its intracellular replication (Figure 1). Such a phenotype of *speG* associated with the intracellular replication of *S*. Typhimurium is significantly expressed after bacterial invasion in cells for a sufficiently long duration (Figures 1B–E) rather than for short durations (Figure 1A), possibly because of the time-dependent accumulation of intracellular metabolites caused by impaired SAT synthesis in the *speG* mutant. Therefore, whether the accumulation of spermidine in *Salmonella* is detrimental to bacterial intracellular proliferation expedited our subsequent quantification of the four major polyamines in *S*. Typhimurium and its *speG*-deleted mutant outside and inside Caco-2 cells.

This study was the first to report that putrescine, spermine, and cadaverine are the polyamines with the highest concentrations and that the concentration of spermidine is the lowest among the four major polyamines in *S*. Typhimurium, regardless of whether *speG* is in the bacterial genome. Our HPLC analysis indicated that putrescine is the predominant polyamine in *S*. Typhimurium SL1344, followed by spermine and cadaverine. The same order was observed in extracellular *S*. Typhimurium Δ*speG*, as well as in intracellular *S*. Typhimurium SL1344 and Δ*speG* in Caco-2 cells (Figure 2). Consistent with our result, a recent study used gas chromatography mass spectrometry and reported that putrescine has the highest positive loading in intracellular profiles of *S*. Typhimurium, just second to succinic acid (Wong et al., 2015). Similar to *E. coli*, putrescine is the most abundant polyamine in *S*. Typhimurium. It is the main constituent of the outer membrane of both bacteria (Koski and Vaara, 1991; Cohen, 1997; Shah and Swiatlo, 2008). Moreover, we observed that *S*. Typhimurium contains a high concentration of spermine and cadaverine, both of which are scarce or absent in *E. coli* (Cohen, 1997; Shah and Swiatlo, 2008). This study was the first to address the relevance of spermine and cadaverine in invasive bacteria such as *Salmonella*. *Shigella* and all enteroinvasive *E. coli* strains lack lysine decarboxylase for the decarboxylation of lysine to synthesize cadaverine, a small polyamine that inhibits the inflammation induced by dysentery bacteria (Casalino et al., 2003). Therefore, the high cellular levels of cadaverine suppressing host inflammation could explain the resistance of *Salmonella* to the intracellular immunity of host cells. Piperidine, a cadaverine metabolite, dose-dependently inhibits the virulence of *S*. Typhimurium during pathogen-host interactions *in vitro* and *in vivo* (Kohler et al., 2002). Further studies must determine how the orchestration of endogenous and exogenous cadaverine modulates the pathogenesis of *Salmonella*. In contrast to the loss of *speG* in *Shigella* inducing the accumulation of intracellular spermidine, which favors bacterial survival under oxidative stress conditions (Campilongo et al., 2014), the intracellular proliferation and survival of *S*. Typhimurium is independent of the accumulation of intracellular spermidine because it has considerably lower concentration of spermidine than of the polyamines in bacterial cells (Figure 2). Furthermore, numerous cellular transport systems can regulate the levels of polyamine in bacteria. *E. coli* exerts polyamine import by the spermidine-preferential PotABCD, putrescine-specific PotFGHI and PuuP transporters (Kashiwagi et al., 1993; Igarashi et al., 2001; Kurihara et al., 2009), putrescine–ornithine exchange by PotE (Kashiwagi et al., 1997), cadaverine–lysine exchange by CadB (Soksawatmaekhin et al., 2004), spermidine excretion by MdtJI (Higashi et al., 2008), and pustrescine export by SapBCDF (Sugiyama et al., 2016). Nevertheless, *speG* did not significantly affect the expression of these genes in *S*. Typhimurium in our study.

*speG*-regulated polyamine synthesis in *S*. Typhimurium varies before and after invasion in human intestinal epithelial cells. Our HPLC analysis indicated that the depletion of *speG* significantly and constantly increased the concentrations of spermine before and after invasion in Caco-2 cells (Figures 2B,D), suggesting that *speG* affects the inhibition of spermine synthesis without being influenced by the intracellular environments of host cells. Contrastingly, the effect of *speG* on intracellular *S*. Typhimurium after 18-h internalization in Caco-2 cells exhibited a different phenotype by modulating the cytoplasmic contents of putrescine, cadaverine, and spermidine, namely the intracellular inhibition of the accumulation of these polyamines. The knockout effect of *speG* on the suppression of the contents of putrescine and cadaverine in extracellular *S*. Typhimurium was the reverse of the enhancement of these polyamines after the invasion of *S*. Typhimurium in Caco-2 cells. *speG* did not influence the concentration of spermidine in extracellular *S*. Typhimurium but significantly increased it in intracellular *S*. Typhimurium. Until now, most studies have investigated polyamines in *E. coli*. In *E. coli*, putrescine is synthesized as in mammalian cells via two pathways, either by the catalyzation of L-ornithine by ornithine decarboxylase (SpeC/SpeF), as in mammalian cells, or by the catalyzation of L-arginine by arginine decarboxylase (SpeA) into agmatine, which is further catalyzed by agmatine ureohydrolase (SpeB). However, spermidine is synthesized from putrescine and decarboxylated S-adenosylmethonine catalyzed by spermidine synthase (SpeE), with the simultaneous metabolization of decarboxylated S-adenosylmethonine to methylthioadenosine. S-adenosylmethonine decarboxylase (SpeD) catalyzes the synthesis of decarboxylated S-adenosylmethonine from S-adenosylmethonine that is originally metabolized from its upstream precursor L-methionine catalyzed by MetK with ATP consumption. Theoretically, the impaired function of SpeG leads directly to the accumulation of spermidine or indirectly to its precursor metabolites, namely putrescine, agmatine, arginine, or ornithine. Unlike the loss of *speG* in *Shigella* that results in higher concentrations of endogenous spermidine (Barbagallo et al., 2011), our study indicated that the genuine *speG* effect is mainly dependent on spermine, rather than on spermidine, in both extracellular and intracellular *S*. Typhimurium, with its versatile modulation on putrescine and cadaverine affected by the intracellular environment of host cells. Considering our finding regarding the effect of *speG* on the intracellular replication of *S*. Typhimurium, we hypothesized that the loss of *speG* could result in the marked accumulation of putrescine, spermine, and cadaverine in intracellular *S*. Typhimurium or affect the expression of other *Salmonella* virulence genes via an unknown pathway to hinder intracellular bacterial replication.

The accumulation of cellular polyamines, including putrescine, spermine, and cadaverine, impedes the intracellular replication of *Salmonella* in human intestinal epithelial cells. Polyamines are indispensable for normal cell growth and affect the stimulation of cell division and proliferation; gene expression for the survival of cells, the regulation of apoptosis, oxidative stress, cell–cell communication, and the synthesis and functions of DNA and protein synthesis, particularly RNA because most of cellular polyamines exist in a polyamine–RNA complex in cells (Igarashi and Kashiwagi, 2010; Lenis et al., 2017). However, studies have rarely determined whether insufficient or excessive polyamines are detrimental to bacterial proliferation. Decreased concentrations of spermidine and putrescine reduce the growth rate of *E. coli* (Cunningham-Rundles and Maas, 1975; Xie et al., 1993). A study reported that exogenous spermine dose-dependently inhibits the *in vitro* growth of *S*. Typhimurium, *E. coli*, and *Staphylococcus aureus*, but not *P. aeruginosa* (Kwon and Lu, 2007). To validate our observation of the increased polyamine contents of non-replicating intracellular *S*. Typhimurium Δ*speG*, we demonstrated that the intracellular proliferation of *S*. Typhimurium was dose-dependently suppressed by putrescine, cadaverine, and spermine in the concentrations estimated from our earlier HPLC analysis (Figure 3). Recent studies have reported several cellular mechanisms controlling the levels of intracellular polyamines fine-tuned by their biosynthesis, catabolism, and transport at the transcription, translation, and protein degradation levels involving feedback loops controlled by polyamine concentrations. These mechanisms include the transcriptional and translational control of ornithine decarboxylase, regulation of polyamine synthesis by antizyme levels, catalyzation of S-adenosylmethionine into decarboxylated S-adenosylmethonine for synthesizing spermidine and spermine from putrescine, controlling polyamine levels through catabolism by oxidation or acetylation to maintain their cellular activity or concentration, and controlling polyamine levels through transport (Filippou et al., 2007; Igarashi and Kashiwagi, 2010; Miller-Fleming et al., 2015; Lenis et al., 2017). Furthermore, the function of polyamines in *S*. Typhimurium had remained largely unknown until the transcriptome of intracellular *S*. Typhimurium revealed the upregulation of genes for putrescine and spermidine biosyntheses during the infection of epithelial cells and macrophages, suggesting an important role of polyamines in bacterial invasion and intracellular survival (Eriksson et al., 2003; Hautefort et al., 2008; Di Martino et al., 2013). A polyamine mutant of *S*. Typhimurium with *speB, speC, speE*, and *speF* deletions exhibited a defective invasion of epithelial cells and attenuation in intracellular replication compared with its wild-type counterpart and the typhoid mouse model (Jelsbak et al., 2012). This defective intracellular replication was enhanced by the complementation of *speB* in the polyamine mutant and exogenous putrescine and spermidine in the culture media before the infection of the cell cultures (Jelsbak et al., 2012). These results indicated a critical role of putrescine and spermidine in controlling virulence in *S*. Typhimurium, most likely through the upregulation of essential virulence loci in SPI-1 and SPI-2 (Jelsbak et al., 2012). However, the genotypes of *speG* in bacterial virulence and their role in regulating the toxicity of excessive polyamines in *Salmonella* remain unclear.

The relationship of polyamines with bacterial flagellation and motility in *Salmonella* has rarely been reported. Polyamines may be involved in the intracellular virulence of *S*. Typhimurium. An early study reported that a housekeeping gene, *sifA*, located within the *potABCD* operon and involved in the periplasmic transport of polyamines, is required for synthesizing *Salmonella*-induced filaments in epithelial cells and for *in vivo* virulence in mice (Stein et al., 1996). Our study demonstrated that *speG* contributes not only to intracellular replication in host cells but also the flagellar biosynthesis and swimming motility of *S*. Typhimurium, suggesting that the *speG*-associated metabolism of polyamines accounts for bacterial morphology and motility and is associated with intracellular growth. In addition, we demonstrated remarkable dose-dependent suppression of *Salmonella* motility by exogenous putrescine, cadaverine, and spermidine. However, *speG* did not significantly affect the expression of other polyamine genes, suggesting that *speG* affects the flagellation of *Salmonella* independently of *speA, speB, speC, speD, speE, cadA*, and *metK*. The production of flagellar protein requires the concomitant synthesis of RNA in *S*. Typhimurium (Aamodt and Eisenstadt, 1968). Because polyamines in a polyamine–RNA complex in cells are essential for RNA synthesis and functions (Igarashi and Kashiwagi, 2010; Lenis et al., 2017), it is reasonably assumed that the unbalanced metabolism of polyamines could interfere with the flagellation of *S*. Typhimurium. A recent proteomic study confirmed the suppression of bacterial flagellation, chemotaxis, SPI-1, TCA cycle, and anaerobic respiration pathways in the metabolic reshuffling of intracellular-replicating *Salmonella* in infected HeLa cells (Liu et al., 2017). Furthermore, the defective intracellular replication of the polyamine mutants with *speB, speC, speE*, and *speF* deletions was in concordance with the downregulation of SPI-1 and SPI-2 genes in *S*. Typhimurium (Jelsbak et al., 2012). Consistently, our microarray study demonstrated the significant downregulation of SPI-1 genes (e.g., *sopD, sopA*, and *sopE2*) and SPI-2 genes (e.g., *sseA, ssaH, sseB*, and *ssaG*) through *speG* deletion in *S*. Typhimurium (Table 2B). Altogether, polyamines are required for the induction of SPI-1 and SPI-2 for *Salmonella* flagellation and motility, and they function as an environmental stimulus to prime *S*. Typhimurium for intracellular proliferation in human epithelial cells.

*speG* can significantly regulate the expression of different genes before and after the internalization of *S*. Typhimurium in human intestinal epithelial cells. For *S*. Typhimurium outside host cells, *speG* is required for the expression of the well-documented genes encoding flagella, fimbria, and T3SS, as well as for the expression of 66 other genes, including those involved in the biosynthesis of inner and outer membranes, bacteriophages, and hydrogenase-1 operon proteins and a few genes encoding hypothetical proteins (Table 2B). Moreover, *speG* can suppress the upregulation of several groups of genes involved in the periplasmic nitrate reductase system (*yojF* and *napF*), glucarate metabolism (e.g., *garL* and *garR*), the phosphotransferase system (SL1344_3736 and SL1344_4467), cytochromes (*cyoABC*), and the succinate reductase complex (*sdhABCD*; Table 2A). These genes have rarely been reported to correlate with the polyamine metabolism and intracellular replication of *Salmonella*. The nap genes encoding periplasmic nitrate reductase can be upregulated under low nitrate conditions from aerobic to anaerobic metabolism to contribute to the luminal growth and virulence of *Salmonella in vivo* (Paiva et al., 2009; Rowley et al., 2012; Lopez et al., 2015). Meanwhile, *speG* could be involved in carbon metabolism for energy because *garL* and *garR* correlate with hydrogen-stimulated carbon acquisition for hydrogen-dependent growth (Lamichhane-Khadka et al., 2011). *speG* modulates SPI-1 virulence because phosphotransferase controls the global transcription regulator Mlc for the complete expression of *hilA, hilD*, and *invF* (Poncet et al., 2009). Similar to the upregulation of *cyoA, cyoB*, and *cyoC* after *speG* deletion in *S*. Typhimurium in our study (Result 3.6), cytochrome *bo* oxidase genes (*cyoA, cyoB*, and *cyoC*) were significantly upregulated on exposure to nitrogen oxide stress, which reduces their vulnerability to oxidative injury (Calderon et al., 2014). Therefore, without *speG* expression, *S*. Typhimurium tends to lose protection against oxidative injury by activating cytochrome *bo* oxidase. In our study, *speG* significantly regulated the expression of four genes encoding the four subunits of the succinate dehydrogenase complex, which is the only enzyme participating in both the electron transport chain and TCA cycle (Oyedotun and Lemire, 2004). Altogether, *speG* can influence a broad spectrum of bacterial metabolisms involving reduction, oxidation, and energy consumption in addition to influencing known virulence factors. In Caco-2 cells, the *ilv*–*leu* operon (*ilvA, ilvC, ilvG, ilvM, leuB, leuC*, and *leuD*) and the histidine operon (*hisC* and *hisG*) were significantly upregulated after *speG* deletion in intracellular *S*. Typhimurium (Table 3A), suggesting the inhibitory function of both operons in the intracellular replication of *Salmonella*. Mutations in *hisG* trigger the intracellular filamentous growth of *S*. Typhimurium and bacterial cell division in eukaryotic host cells (Henry et al., 2005), which is in concordance with our observation of *hisG* upregulation in the poorly proliferating *speG* mutant of intracellular *S*. Typhimurium in Caco-2 cells.

In conclusion, *speG* is required for the intracellular replication of *S*. Typhimurium and is dependent on the duration after bacterial invasion and independent on cell types. Putrescine, spermine, and cadaverine are the predominant polyamines in *S*. Typhimurium. The effects of *speG* on the biosynthesis of these polyamines vary outside and inside host cells and affect the expression of previously reported and unreported *Salmonella* virulence genes involved in a broad spectrum of cellular functions rather than other polyamine-associated genes. Thus, *speG* plays an independent key role in the polyamine metabolism and virulence regulation of *Salmonella*.

## Ethics statement

This study was approved by Taipei Medical University-Joint Institutional Review Board (No. 201205007). Neither human participants nor animals were involved in this study.

## Author contributions

S-BF designed the research and wrote the article; C-JH, C-HH, K-CW, N-WC, and H-YP performed the experiments and analyzed the data; S-BF, H-WF, M-TH, and C-KC conceived the experiments and contributed reagents, materials, and analysis tools.

### Conflict of interest statement

The authors declare that the research was conducted in the absence of any commercial or financial relationships that could be construed as a potential conflict of interest.

## Supplementary material

Supplemetary Figure 1 The HPLC chromatogram of the mixed four diluted polyamine standards.

Supplemetary Figure 2 The regression equations derived from the five dilutions of four polyamine standards.

Data Sheet 1 The 29 significantly upregulated genes **(A)** and the 110 significantly downregulated genes **(B)** of the S. Typhimurium Δ*speG* mutant compared with its parental wild-type strain SL1344 (in the order of log2 fold change).

Data Sheet 2 The 1,964 upregulated genes **(A)** and 2,664 downregulated genes **(B)** of the intracellular *S*. Typhimurium Δ*speG* mutant compared with the intracellular *S*. Typhimurium SL1344 after their invasion in Caco-2 cells for 18 h (in the order of log2 fold change).

## Abbreviations

ATP: adenosine triphosphate
cDNA: complementary deoxyribonucleic acid
CFU: colony-forming units
DMEM: Dulbecco's modified Eagle medium
DNA: deoxyribonucleic acid
*E. coli*: *Escherichia coli*
FBS: fetal bovine serum
HEPES: 4-(2-hydroxyethyl)piperazine-1-ethanesulfonic acid
HPLC: high-performance liquid chromatography
LB: Luria–Bertani
MOI: multiplicity of infection
mRNA: messenger ribonucleic acid
PBS: phosphate-buffered saline
PCR: polymerase chain reaction
qRT-PCR: quantitative real-time polymerase chain reaction
RNA: ribonucleic acid
*S*. Typhimurium: *Salmonella enterica* serovar Typhimurium
SAT: spermidine acetyltransferase
SPI: *Salmonella* pathogenicity island
T1SS: type I secretion system
T3SS: type III secretion system
TCA: trichloroacetic acid.
