# Supplementary figures and images for: speG Is Required for Intracellular Replication of Salmonella in Various Human Cells and Affects Its Polyamine Metabolism and Global Transcriptomes
Source: Front Microbiol. 2017 Nov 15;8:2245. doi: 10.3389/fmicb.2017.02245 (PMC5694781; doi:10.3389/fmicb.2017.02245)

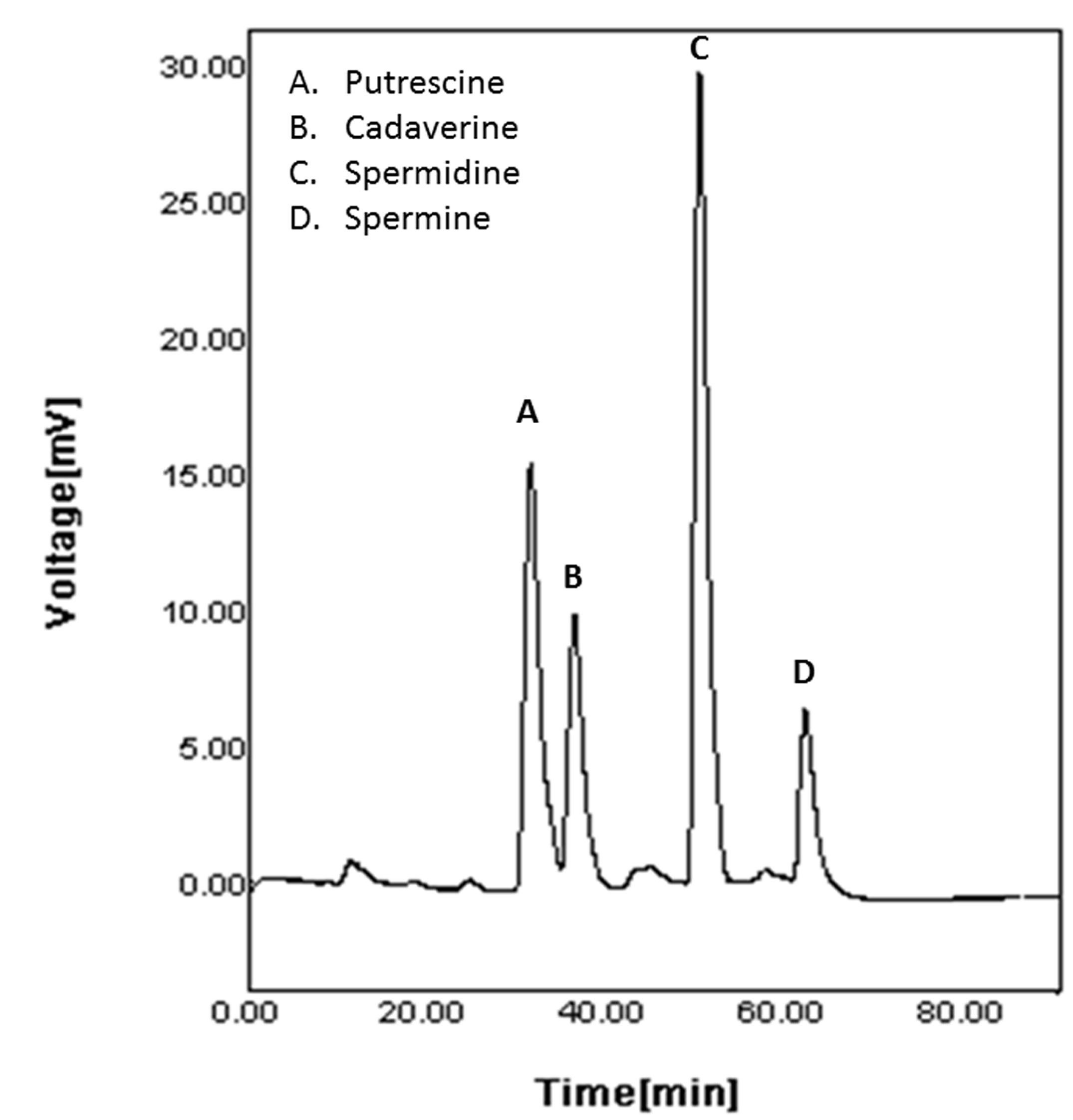

Supplement: Supplemetary Figure 1 — The HPLC chromatogram of the mixed four diluted polyamine standards. [file Image1.TIF]

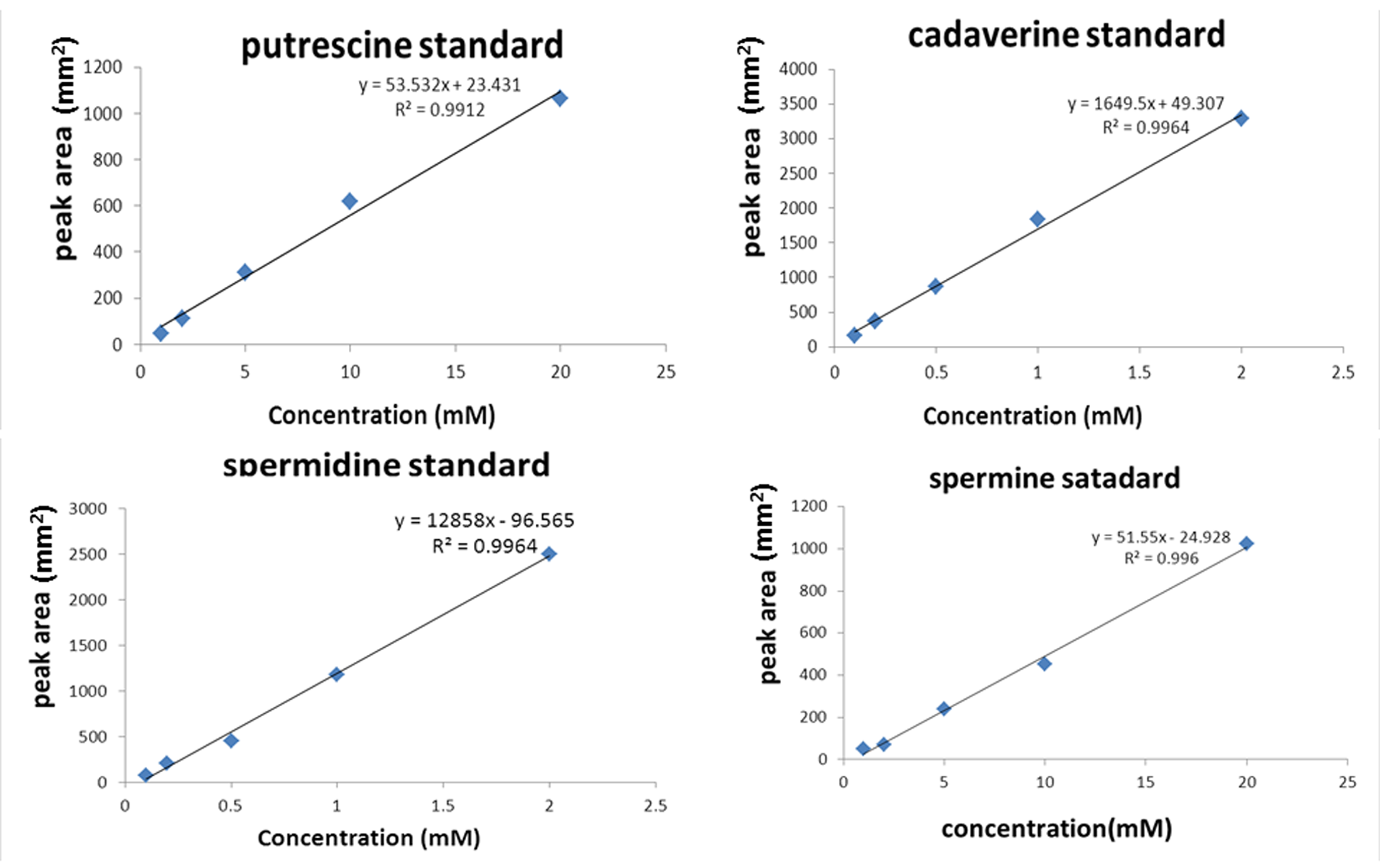

Supplement: Supplemetary Figure 2 — The regression equations derived from the five dilutions of four polyamine standards. [file Image2.TIF]
